# POLE3-POLE4 Is a Histone H3-H4 Chaperone that Maintains Chromatin Integrity during DNA Replication

**DOI:** 10.1016/j.molcel.2018.08.043

**Published:** 2018-10-04

**Authors:** Roberto Bellelli, Ondrej Belan, Valerie E. Pye, Camille Clement, Sarah L. Maslen, J. Mark Skehel, Peter Cherepanov, Genevieve Almouzni, Simon J. Boulton

**Affiliations:** 1 The Francis Crick Institute, 1 Midland Road, London NW1 1AT, UK; 2 Institut Curie, PSL Research University, CNRS, UMR3664, Equipe Labellisée Ligue contre le Cancer, Paris, France; 3 Sorbonne Universités, UPMC Univ Paris 06, CNRS, UMR3664, Paris, France; 4 MRC Laboratory of Molecular Biology, Francis Crick Avenue, Cambridge Biomedical Campus, Cambridge CB2 0QH, UK

**Keywords:** POLE3-POLE4 complex, replication-coupled nucleosome assembly, histone chaperone, epigenome stability

## Abstract

Maintenance of epigenetic integrity relies on coordinated recycling and partitioning of parental histones and deposition of newly synthesized histones during DNA replication. This process depends upon a poorly characterized network of histone chaperones, remodelers, and binding proteins. Here we implicate the POLE3-POLE4 subcomplex of the leading-strand polymerase, Polε, in replication-coupled nucleosome assembly through its ability to selectively bind to histones H3-H4. Using hydrogen/deuterium exchange mass spectrometry and physical mapping, we define minimal domains necessary for interaction between POLE3-POLE4 and histones H3-H4. Biochemical analyses establish that POLE3-POLE4 is a histone chaperone that promotes tetrasome formation and DNA supercoiling *in vitro*. In cells, POLE3-POLE4 binds both newly synthesized and parental histones, and its depletion hinders helicase unwinding and chromatin PCNA unloading and compromises coordinated parental histone retention and new histone deposition. Collectively, our study reveals that POLE3-POLE4 possesses intrinsic H3-H4 chaperone activity, which facilitates faithful nucleosome dynamics at the replication fork.

## Highlights

• Mammalian POLE3-POLE4 is a bona fide H2A-H2B histone fold complex
• The POLE3-POLE4 complex binds to histones H3-H4 *in vitro* and *in vivo*
• POLE3-POLE4 induces tetrasome formation and DNA supercoiling *in vitro*
• POLE3-POLE4 chaperone activity promotes chromatin integrity in mammalian cells

## Graphical Abstract

**Figure:**
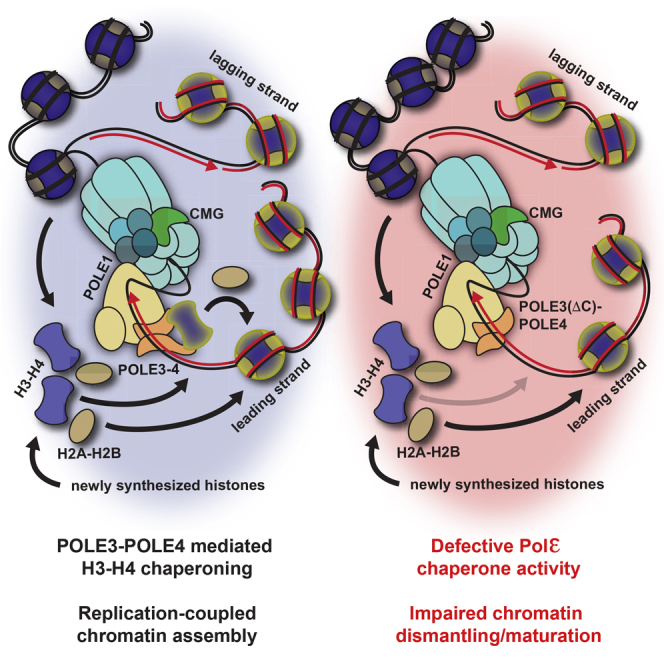

## Teaser

Bellelli et al. report that mammalian POLE3-POLE4 binds to histones H3-H4 and promotes tetrasome formation and DNA supercoiling, acting as a bona fide histone chaperone. Consistent with this, cells lacking POLE3/POLE4 exhibit defective RPA accumulation and PCNA retention on chromatin, features of defective chromatin dismantling/maturation at the replication fork.

## Introduction

During S phase of the cell cycle, accurate and processive replication of genomic DNA has to be coupled to duplication of the epigenetic information encoded in histones and their post-translational modifications (Kouzarides, 2007). For this to happen, chromatin must be disrupted ahead of the replication fork and restored in a timely and regulated fashion on sister chromatids, a process known as replication-coupled nucleosome assembly (Groth et al., 2007a, Alabert and Groth, 2012, Almouzni and Cedar, 2016). This process relies on two different but interlocked mechanisms involving the recycling of parental histones and the deposition of newly synthesized ones.

Coordination of these processes is particularly important for both the transmission of heterochromatic domains, such as those next to centromeres and telomeres, and the maintenance of cellular differentiation and identity (O’Sullivan et al., 2014, Cheloufi et al., 2015, Müller and Almouzni, 2017). Furthermore, alteration of chromatin assembly has been linked to human genetic diseases, such as Wolf-Hirschhorn syndrome and congenital dyserythropoietic anemia type I (Ask et al., 2012, Kerzendorfer et al., 2012), as well as to acquired diseases such as cancer (Corpet et al., 2011). Changes in chromatin composition and structure also impact on aging (Feser et al., 2010, O’Sullivan et al., 2010).

The mechanisms that coordinate parental histone recycling and deposition of newly synthesized histones and how these function during leading- and lagging-strand replication remain to be deciphered. Several histone chaperones have been implicated in handling histones and participate in their transfer during DNA replication (De Koning et al., 2007). The histone chaperone CAF-1 (chromatin assembly factor 1) deposits new histones in a DNA synthesis-dependent manner (Smith and Stillman, 1989, Shibahara and Stillman, 1999, Verreault et al., 1996). With respect to recycling of parental histones, a role has been recently ascribed to the MCM2 component of the eukaryotic replicative helicase MCM2-7, which directly binds histones H3-H4 via a N-terminal domain (Ishimi et al., 2001, Foltman et al., 2013), together with the essential histone chaperone Asf1 (Groth et al., 2007b). Structural studies have revealed that MCM2 acts by shielding H3-H4 surfaces normally bound by DNA or histones H2A/H2B (Huang et al., 2015, Richet et al., 2015). The histone chaperone FACT (facilitates chromatin transcription), which is able to chaperone both H2A/H2B and H3/H4 histones, has been found to travel with the replisome in *S. cerevisiae* (Gambus et al., 2006) and is proposed to participate in chromatin dismantling/deposition at the fork (Foltman et al., 2013, Yang et al., 2016, Kurat et al., 2017). Recent studies have also shown that the single-strand binding protein RPA binds histones H3-H4 and promotes nucleosome assembly at the replication fork (Liu et al., 2017).

Observations in budding and fission yeast have suggested a role for the leading-strand polymerase Polε in maintaining heterochromatin regions (Iida and Araki, 2004, Li et al., 2011); this function seems to be mainly dependent upon the smallest subunits of Polε, Dpb3 and Dpb4 (Iida and Araki, 2004, He et al., 2017). ΔDpb3 and ΔDpb4 yeast strains exhibit defective heterochromatin maintenance, which is also shared with strains lacking essential replisome-associated chaperones such as CAF-1, Asf1, and MCM2 HBD (histone binding domain) (Singer et al., 1998, Kaufman et al., 1997, Foltman et al., 2013). How Dpb3 and Dpb4 contribute to the maintenance of heterochromatin remains unclear.

Here we show that the POLE3-POLE4 accessory subunits of mammalian Polε selectively bind to histones H3-H4 during replication-coupled nucleosome assembly. We define the mechanistic basis of POLE3-POLE4 binding and uncover an intrinsic chaperone activity toward H3-H4. In mammalian cells, depletion of POLE3 or POLE4 or removal of the C terminus of POLE3, which confers binding to H3-H4, directly impacts on nucleosome dynamics at the replication fork. Collectively, our work reveals mammalian POLE3-POLE4 as a replisome-associated histone H3-H4 chaperone that plays an important role in chromatin maintenance during DNA replication.

## Results

### The POLE3-POLE4 Complex Is a Bona Fide H2A-H2B Histone Fold Motif Complex

POLE4, the smallest accessory subunit of Polε, is required to maintain the stability of the whole Polε complex in mice (Bellelli et al., 2018). Indeed, *Pole4*^−/−^ mouse cells or knockdown of POLE4 in human cells drastically reduced the levels of POLE3, which suggests that POLE3 and POLE4 might form a stable subcomplex *in vivo* (Bellelli et al., 2018). To test this hypothesis, we performed a pull-down assay with purified human POLE3 and POLE4 *in vitro*. GST-tagged POLE3 and POLE4 proteins interact with His-tagged POLE4 and POLE3, respectively, and form a stable complex at both physiological (150 mM NaCl) and high (1 M NaCl) salt concentrations (Figures S1A and S1B).

**Figure 1:**
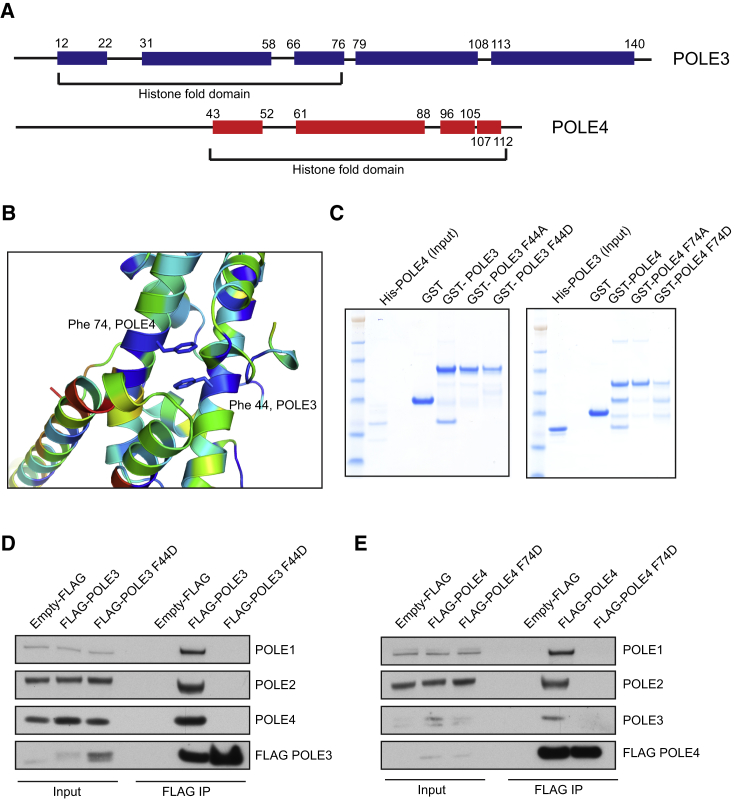
The POLE3-POLE4 Complex Is a Bona Fide H2A-H2B Histone Fold Complex (A) Cartoon depicting human POLE3 and POLE4 proteins and their predicted structural motifs. α helices are represented as squared boxes, while histone fold motifs are indicated by brackets. (B) Modeled POLE3-POLE4 dimer with conserved Phe44 and Phe74 in stick format. Consurf was used to color residues by conservation from blue (most conserved) to red (least conserved). (C) *In vitro* GST pull-downs of the indicated GST-tagged proteins in the presence of His-POLE4 (left) or His-POLE3 (right). (D) Western blot analysis of FLAG IPs from whole-cell extracts of HeLa TRex-expressing empty FLAG, FLAG-POLE3 WT, or F44D mutant. (E) Western blot analysis of FLAG IPs from whole-cell extracts of HeLa TRex-expressing empty FLAG, FLAG-POLE4 WT, or F74D mutant.

POLE3 contains a histone fold domain (H2B-like) located at its N terminus, followed by two α helices, while POLE4 consists of a C-terminal H2A-like histone fold domain, preceded by a predicted flexible and unstructured tail (Figure 1A). As POLE3 and POLE4 possess histone folds that resemble those of H2A or H2B (Gnesutta et al., 2013), a structural model of POLE3 was superposed onto H2B and POLE4 onto H2A, using Phyre2 (Kelley et al., 2015) and a defined nucleosome structure as a template (PDB: 1s32). The resulting structural model almost completely superimposes with the recently published crystal structure of yeast Dpb3-Dpb4 (He et al., 2017) (Figure S1D). The modeled POLE3-POLE4 dimer revealed conserved residues Phe44 in POLE3 and Phe74 in POLE4, located in the central α helix (α2) of their respective histone folds, as having high potential to interact via pi stacking of their side chains (Figures 1B and S1C). As shown in Figure 1C, mutation of Phe44 in POLE3 and Phe74 in POLE4 to alanine or aspartic acid, respectively, was sufficient to abolish interaction between POLE3 and POLE4, validating our structural model.

Next, we generated stable human cell lines expressing FLAG-tagged POLE3 WT (wild-type) or F44D and POLE4 WT or F74D under a tetracycline-regulated promoter (HeLa TRex) and analyzed interactions between WT and mutant proteins as well as with other components of the Polε complex. In agreement with our *in vitro* data, we failed to observe endogenous POLE4 in FLAG immunoprecipitates (IPs) from extracts of FLAG-POLE3 F44D-expressing cells (Figure 1D). Conversely, an interaction between endogenous POLE3 and exogenously expressed FLAG-POLE4 F74D was not observed (Figure 1E). Importantly, abrogation of this interaction led to complete loss of the other components of the Polε complex, POLE1 and POLE2, in FLAG IPs (Figures 1D and 1E), which indicates that interaction between POLE3 and POLE4 is crucial for their association with the Polε catalytic core.

### The POLE3-POLE4 Complex Interacts *In Vitro* with H3-H4

Based on the results of mass spectrometric analysis of Dpb4 and Dpb3 (yeast homologs of POLE3 and POLE4) (Tackett et al., 2005, He et al., 2017) and their involvement in maintaining heterochromatin (Iida and Araki, 2004), we hypothesized that POLE3-POLE4 might directly interact with histones, connecting the replisome and the histone assembly pathways at the replication fork.

**Figure 2:**
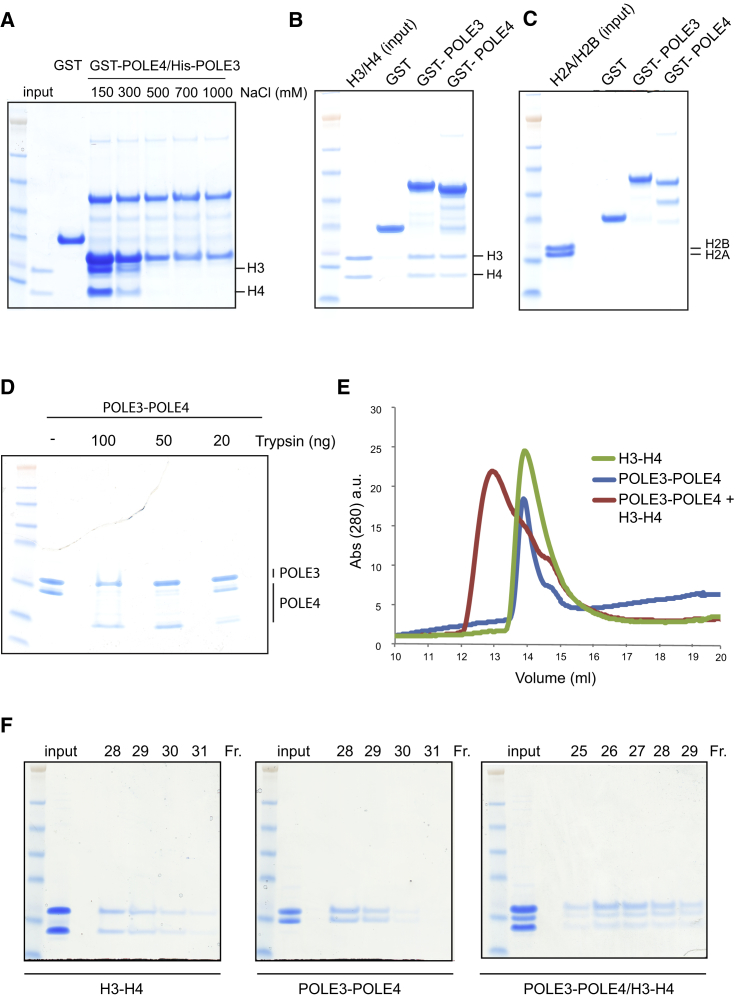
The POLE3-POLE4 Complex Interacts *In Vitro* with H3-H4 (A) *In vitro* GST pull-down of the GST-POLE4/His-POLE3 complex in the presence of H3-H4 at the described NaCl concentrations. (B) *In vitro* GST pull-down of the indicated GST-tagged proteins in the presence of H3-H4. Experiments were performed in 150 mM NaCl. (C) *In vitro* GST pull-down of the indicated GST-tagged proteins in the presence of H2A-H2B dimers. Experiments were performed in 150 mM NaCl. (D) Limited trypsin digestion of recombinant POLE3-POLE4 complex in the presence of the indicated trypsin concentrations. (E) Analytical gel filtration of POLE3-POLE4, H3-H4, and POLE3-POLE4-H3-H4 complexes in 300 mM NaCl concentrations. (F) SDS-PAGE analysis of gel filtration fractions from (E).

POLE3 and POLE4, as part of the Polε complex, are considered to be constitutive components of the replicative helicase CMG (CDC45/MCM2-7/GINS1-4) (Moyer et al., 2006, Sun et al., 2015), which is known to interact with H3-H4 via its MCM2 subunit (Ishimi et al., 2001, Foltman et al., 2013). To exclude indirect interactions via MCMs and/or other components of the CMG, we tested a possible interaction between POLE3-POLE4 and H3-H4, the most likely nucleosome intermediate interactor based on our structural modeling. To this aim, we generated a tagged POLE3-POLE4 complex (GST-Pole4/His-POLE3) and examined binding to purified H3-H4 histones at different salt concentrations. The POLE3-POLE4 complex was able to strongly pull down H3-H4 in a salt-dependent manner; interaction was maximal at 150 mM NaCl, was reduced to ∼50% at 300 mM, and was almost undetectable at 500 mM NaCl, a salt concentration at which the POLE3-POLE4 complex remains stable (Figure 2A). Surprisingly, either POLE3 or POLE4 alone was able to efficiently pull down H3-H4 at physiological salt concentration (150 mM NaCl), suggesting that this interaction might involve several domains of the two subunits (Figures 2B and S2A). Similar results were obtained with His-tagged POLE3 and POLE4 (Figure S2B). Of note, POLE3 and POLE4 interacted with the replicative H3.2-H4 isoform in a similar manner, excluding a possible specific affinity toward the H3.3 variant, which we initially used to test interactions (Figures 2A and 2B) and that is deposited in a replication-independent manner (Tagami et al., 2004) (Figure S2C). Finally, neither POLE3 nor POLE4 was able to bind H2A and H2B under the same experimental conditions, suggesting a specific interaction between POLE3-POLE4 and H3-H4 (Figure 2C).

To further characterize this interaction, we generated an untagged POLE3-POLE4 complex by a three-step purification protocol involving GST affinity chromatography, GST tag removal, heparin column, and size-exclusion chromatography (Figure S2D). Limited proteolysis of POLE3-POLE4 by trypsin digestion showed that the C-terminal portion of POLE3, which is located outside its histone fold motif, is likely structured and protected, suggesting an unexpected conformation of this subunit (Fontana et al., 2004). Conversely, the N-terminal tail region of POLE4 is likely extended and flexible, in accordance with structural predictions. Indeed, POLE4 undergoes rapid trypsin digestion, as shown by SDS-PAGE analysis and western blotting (Figures 2D and S2E). Analytical size-exclusion chromatography analysis of POLE3-POLE4 incubated with H3-H4 revealed a co-complex of POLE3-POLE4/H3-H4 in 300 mM NaCl, as shown by the different elution peaks of the three complexes (POLE3-POLE4, H3-H4, and POLE3-POLE4-H3-H4) and the SDS-PAGE analysis of the fractions (Figures 2E and 2F). Size-exclusion chromatography analysis of the POLE3-POLE4/H3-H4 co-complex at a lower salt concentration (150 mM NaCl) proved to be extremely difficult due to histone aggregation on gel filtration columns, as previously reported (e.g., Richet et al., 2015).

### H/D Exchange Mass Spectrometry Reveals Conformational Changes and Interaction Domains Involved in the Interaction between POLE3-POLE4 and H3-H4

**Figure 3:**
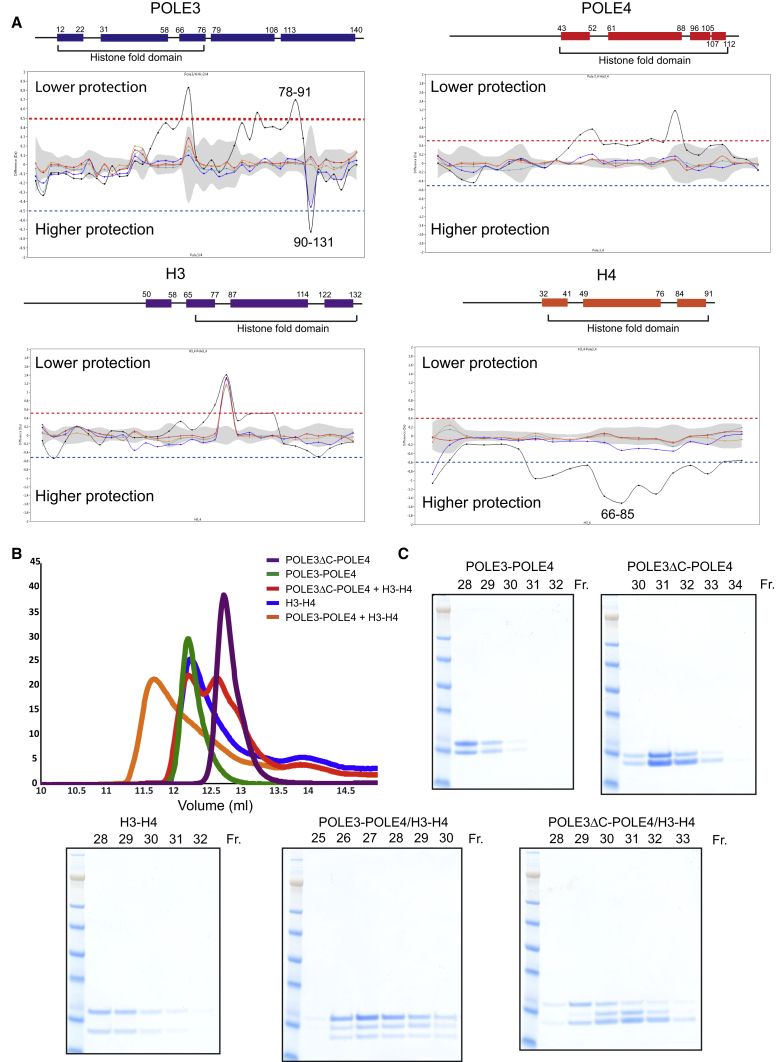
H/D Exchange Mass Spectrometry and Protein Pull-Downs Identify Interaction Domains between POLE3-POLE4 and H3-H4 Complexes (A) Difference plot of hydrogen/deuterium (H/D) exchange data from POLE3 (upper left), POLE4 (upper right), H3 (lower left), and H4 (lower right). A cartoon depicting the domains of the analyzed proteins is shown on the top of the graph. Experimental error is reported in gray, while different colors represent different D_2_O incubation times. More exposed and protected regions are located, respectively, on the higher and lower part of the graph. Peptide aa numbers from highly protected or exposed regions of POLE3 and H4 are shown. ±0.5 Dalton difference is indicated with red/blue lines, which represents 98% confidence limit, so that peptides above or below these lines can be considered significantly changed. Difference heatmaps are provided in Figure S3A. (B) Analytical gel filtration of the described protein complexes in 300 mM NaCl concentrations. (C) SDS-PAGE analysis of gel filtration fractions from the described protein complexes.

In an attempt to define the interaction surfaces between POLE3-POLE4 and H3-H4, we conducted hydrogen/deuterium (H/D) exchange mass spectrometry of POLE3-POLE4 and H3-H4 complexes alone or in combination (Liu et al., 2016, Mattiroli et al., 2017). Deuterium labeling was conducted in triplicate at 23°C, in 300 mM NaCl, at five time points (0.3, 3, 30, 300, and 3,000 s) followed by quenching, pepsin digestion, and liquid chromatography-mass spectrometry. A total of 120 peptides were identified with a sequence coverage of 70%–99%. A difference plot was generated by subtracting deuterium incorporation for the same identified peptide upon incubation of POLE3-POLE4 with or without H3-H4. Peptides were plotted on the x axis from N to C terminus, with the y axis showing the difference in Daltons (Figure 3A). Both POLE3-POLE4 and H3-H4 showed several regions of change in solvent exposure upon complex formation. As shown in Figures 3A and S3A, H/D exchange mass spectrometry revealed several regions with increased or decreased solvent accessibility, the latter likely to represent the major protein-protein interaction surfaces. Two major regions of decreased H/D exchange were evident, corresponding to the C-terminal portion of POLE3 (aa 90–131) and a region extending between aa 68 and 85 of H4, mostly spanning through α2 and the second loop (L2) of the histone fold domain (Figures 3A and S3A). H3 showed only minimal protection toward the N- and C-terminal regions and decreased protection in the central portion (aa 84–99), which may represent a conformational change upon H4 engagement. Similarly, POLE3 also showed an area of exposure (aa 87–91) that might result from the binding of its C-terminal region to H4.

The H/D exchange data raised the possibility that the major interaction between the two complexes involves the C terminus of POLE3 and the histone fold domain of H4. Indeed, the last α helix of POLE3, corresponding to aa 113–140, was sufficient to pull down H3-H4, while the histone fold domain was dispensable (Figure S4A). Conversely, the histone fold domain of POLE4, which showed several areas of change in solvent accessibility upon interaction with H3-H4, was the only portion of POLE4 important for the interaction (Figure S4B). These data suggest that a POLE3-POLE4 complex, lacking the C-terminal portion of POLE3, might represent a separation-of-function mutant, potentially lacking significant interaction with histones H3-H4 while retaining interaction with Polε complex components.

To test this hypothesis, we generated an untagged POLE3-POLE4 complex lacking the last α helix (aa 113–140, Figure S4A) of POLE3 (POLE3ΔC-POLE4) and subjected it to size-exclusion chromatography analysis in the presence or absence of histones H3-H4. In contrast to WT POLE3-POLE4, we observed two distinct peaks on gel filtration when the POLE3ΔC-POLE4 complex was incubated with H3-H4 and no significant shifts, suggesting the absence of stable complex formation (Figure 3B). We also performed GST pull-down of histones H3-H4 using a complex of GST-tagged POLE3 WT or ΔC and His-tagged POLE4. In contrast to WT POLE3-POLE4, interaction of POLE3ΔC-POLE4 with H3-H4 was almost undetectable (see Figure S4C), suggesting that the C-terminal portion of POLE3 is sufficient and essential for interaction with H3-H4.

To identify the regions of H3-H4 involved in complex formation with POLE3-POLE4, we purified H3 and H4 proteins lacking their N-terminal tails (H3-H4Δtails) and refolded them into H3-H4 tetramers. Despite lower solubility and a tendency to precipitate during refolding, H3-H4Δtails retained the ability to interact with POLE3 and POLE4, while biotinylated H3 and H4 tail peptides alone failed to pull down the POLE3-POLE4 complex (Figures S4D and S4E). Conversely, GST-POLE3 and POLE4 were unable to interact with H3 and H4 tail peptides in reciprocal experiments (Figures S4F and S4G). These results indicate that the histone tails are dispensable for POLE3-POLE4 binding.

### The POLE3-POLE4 Complex Promotes Tetrasome Formation and Plasmid Supercoiling *In Vitro*

**Figure 4:**
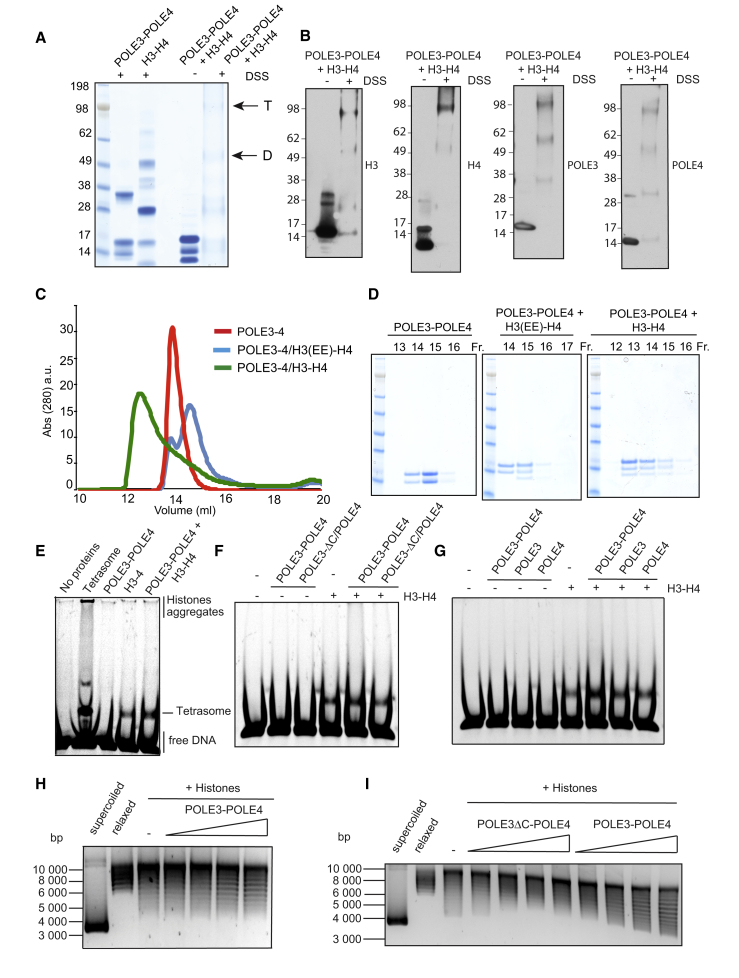
The POLE3-POLE4 Complex Binds to H3-H4 Dimers and Tetramers and Promotes Tetrasome Formation and Supercoiling *In Vitro* (A) DSS crosslinking experiments performed in 150 mM NaCl. Lane 4 shows control without crosslinking; lanes 1, 2, and 5 show samples incubated with 1 mM DSS for 30 min at 23°C and resolved by SDS-PAGE and Coomassie staining. Lane 3 was left empty. Two black arrows indicate the molecular weight species identified upon crosslinking of POLE3-POLE4/H3-H4 co-complex. T (tetramers) indicates POLE3-POLE4 binding to H3-H4 tetramers, while D (dimers) indicates POLE3-POLE4 binding to dimers. (B) Western blot analysis of DSS crosslinking experiments performed in 150 mM NaCl. Lane 1 shows control without crosslinking; lane 2 shows sample incubated with 1 mM DSS for 30 min at 23°C. Samples were resolved by SDS-PAGE, transferred to nitrocellulose membranes, and incubated with α-H3, α-H4, α-POLE3, and α-POLE4 antibodies, from left to right. (C) Analytical gel filtration of POLE3-POLE4 complex alone or in combination with H3-H4 or H3(EE)-H4. Experiments were performed in 300 mM NaCl to prevent histone aggregation. (D) SDS-PAGE analysis and Coomassie staining of gel filtration fractions from (C). (E) Tetrasome assembly on linear DNA (Widom 601 sequence) monitored by native PAGE. Lane 2 shows tetrasome preassembled by salt dilution method; lanes 3–5 show linear DNA incubated with the indicated proteins. (F and G) Tetrasome assembly on linear DNA, performed as in (E), with the indicated proteins. (H) Plasmid supercoiling assay resolved by native agarose gel electrophoresis. Lane 1 shows supercoiled control phix174 RF1 DNA; lane 2, phix174 RF1 DNA relaxed by TOPO I; and lanes 3–7, phix174 RF1 DNA incubated, in the presence of TOPO I, with histones and increasing concentrations of POLE3-POLE4. (I) Plasmid supercoiling assay performed as described in (H) using increasing concentrations of POLE3ΔC-POLE4 or POLE3-POLE4.

Histones H3 and H4 can exist as both dimers and tetramers in solution, depending on salt concentration. To determine whether POLE3-POLE4 binds to H3 and H4 dimers or tetramers, we performed crosslinking experiments as previously described to assess MCM2 in complex with H3-H4 (Huang et al., 2015). To this aim, we incubated POLE3-POLE4 and H3-H4 alone or in combination with the crosslinker DSS (disuccinimidyl suberate). After quenching, we resolved crosslinked complexes by SDS-PAGE, which revealed the presence of two major bands: a higher one corresponding to a POLE3-POLE4 complex binding H3-H4 in a tetrameric conformation (predicted MW ∼83 kDa) and an additional lower-molecular-weight species corresponding to POLE3-POLE4 binding to H3-H4 dimers (predicted MW ∼56 kDa) (Figure 4A). Western blotting using anti-H3, -H4, -POLE3, and -POLE4 antibodies confirmed this hypothesis, suggesting that POLE3-POLE4 can bind to both H3-H4 dimers and tetramers under physiological salt concentrations (Figure 4B) and at 300 mM NaCl (Figure S5A).

To explore the possibility that POLE3-POLE4 preferentially binds to tetramers, we incubated the POLE3-POLE4 complex with a tetramerization disruption mutant of H3 (L126R I130E, hereafter referred to as H3EE) (Huang et al., 2015). Analytical size-exclusion chromatography at 300 mM NaCl revealed the presence of two distinct peaks when POLE3-POLE4 was incubated with H3(EE)-H4 (but not H3-H4 WT), suggesting that, at least under these conditions, stable complex formation is prohibited (Figures 4C and 4D). At 150 mM NaCl, a GST-POLE3/His-POLE4 complex was unable to stably bind H3(EE)-H4 (Figure S5B). Similar results were obtained using a single point mutant of H3 (H3 C110E), which targets a different residue of H3 and also prevents H3-H4 tetramerization (Bowman et al., 2011) (Figure S5C).

To examine the possibility that the POLE3-POLE4 complex acts as a bona fide H3-H4 chaperone, we tested if POLE3-POLE4 could assemble histones H3-H4 onto linear DNA and relax circular plasmid DNA *in vitro*, all general features of histone chaperones (Hammond et al., 2017). As shown in Figure 4E, the POLE3-POLE4 complex, when incubated with H3-H4 and a linear DNA substrate (Lowary and Widom, 1998), increased the formation of tetrasomes in a concentration-dependent manner (Figures 4E and S5D). Importantly, the POLE3ΔC-POLE4 complex, despite being able to bind DNA *in vitro* (Figure S5E), did not show activity in tetrasome formation assays (Figure 4F). Furthermore, although POLE3 and POLE4 can bind H3 and H4 individually *in vitro*, they were unable to stimulate tetrasome formation alone, indicating that the full-length POLE3-POLE4 complex is needed for histone chaperone activity (Figure 4G). Lastly, we tested the capacity of POLE3-POLE4, in the presence of H3-H4 and topoisomerase 1, to induce histone deposition as assessed in a plasmid supercoiling assay (Germond et al., 1975). Importantly, the POLE3-POLE4 complex alone showed no significant supercoiling activity (Figure S5F). However, addition of increasing concentrations of POLE3-POLE4 to H3-H4 progressively increased supercoiling, suggesting that the POLE3-POLE4 complex is indeed able to promote H3-H4 deposition and DNA supercoiling (Figures 4H and S5G). Importantly, the POLE3ΔC-POLE4 mutant, which is compromised for H3-H4 binding, failed to induce supercoiling *in vitro* (Figure 4I). These data reveal that POLE3-POLE4 possesses intrinsic histone chaperone toward H3-H4 (Figures 4H and 4I).

### The POLE3-POLE4 Complex Interacts with H3-H4 *In Vivo*

**Figure 5:**
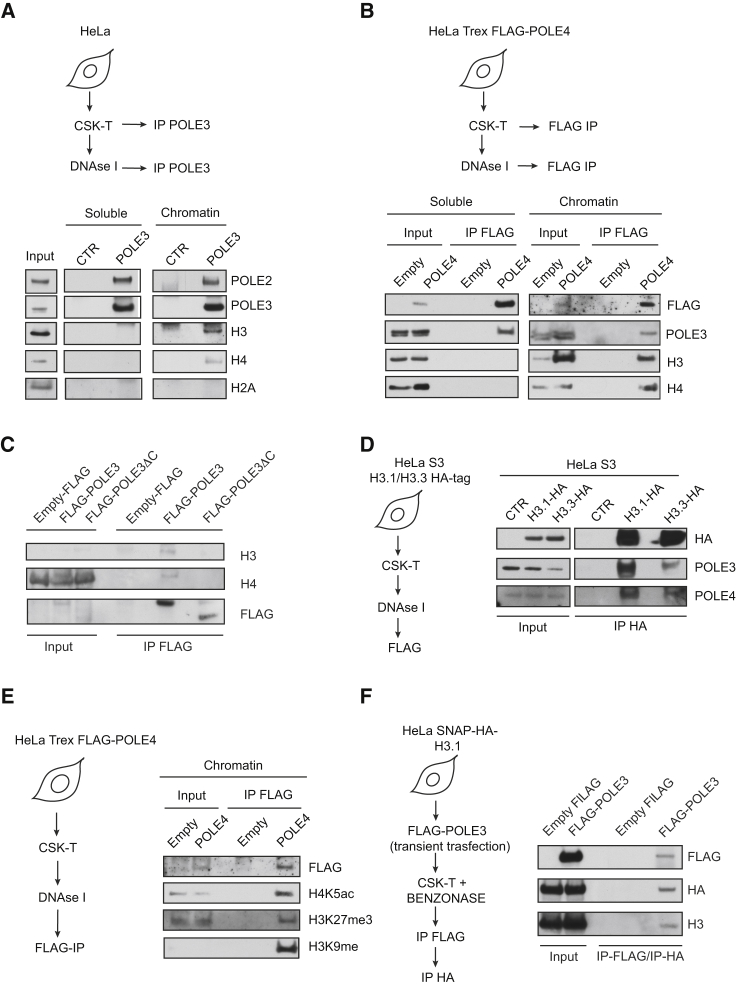
POLE3 and POLE4 Interact with H3-H4 *In Vivo* (A) IP of endogenous POLE3 from human HeLa cells performed after CSK-Triton (0.5%) extraction on soluble and DNaseI-digested chromatin fractions. After SDS-PAGE, western blotting was performed using antibodies against the indicated proteins. (B) FLAG IP experiments from HeLa TRex-expressing empty FLAG or FLAG-POLE4 under tetracycline-regulated promoter. Cells were induced with doxycycline for 24 hr and lysed in CSK-Triton 0.5%. FLAG IPs were performed on soluble and DNaseI-digested chromatin fractions. After SDS-PAGE and nitrocellulose transfer, membranes were incubated with antibodies against the indicated proteins. (C) FLAG IP experiments from HeLa TRex-expressing empty FLAG, FLAG-POLE3 WT, or POLE3ΔC mutants under a tetracycline-regulated promoter. Cells induced with doxycycline for 24 hr were lysed in CSK-Triton 0.5%, and FLAG IP was performed on DNaseI-digested chromatin. After SDS-PAGE, western blotting was performed using antibodies against the indicated proteins. (D) HA tag IP experiments from HeLa S3 cells stably expressing HA-H3.1 and HA-H3.3 from soluble and DNaseI-digested chromatin fractions. After SDS-PAGE, western blotting was performed using antibodies against HA tag, POLE3, and POLE4. (E) FLAG IPs from HeLa TRex-expressing empty FLAG or POLE4-FLAG mutants under tetracycline-regulated promoter. Cells were induced with doxycycline for 24 hr and lysed in CSK-Triton 0.5%. FLAG IPs were performed on DNaseI-digested chromatin fractions; after SDS-PAGE and western blotting, membranes were incubated with antibodies against marks specific of newly synthesized or parental histones. (F) Sequential IP experiments performed on empty FLAG or FLAG-POLE3 HeLa SNAP-HA-H3.1-transfected cells. After cell lysis in CSK-Triton 0.5%, chromatin was solubilized with benzonase and incubated with anti-FLAG agarose beads. After subsequent FLAG bead elution in 3xFLAG peptides (1 mg/mL concentration), HA IPs were performed, followed by SDS-PAGE and western blotting using antibodies against the indicated proteins.

To analyze the interaction between the POLE3-POLE4 complex and H3-H4 *in vivo*, we IPed endogenous POLE3 from CSK-extracted soluble fractions and DNaseI-digested chromatin from human HeLa cells and analyzed interactions with histones and other Polε components (Groth et al., 2007b). As shown in Figure 5A, we failed to detect histones in POLE3 IPs from the soluble fraction. However, POLE3 IPed POLE2 and H3-H4, but not H2A, from DNaseI-digested chromatin. These data suggest that POLE3-POLE4 might chaperone H3-H4 on chromatin during nucleosome processing at the replication fork.

To exclude the possibility that the interaction between POLE3-POLE4 and H3-H4 is CHRAC dependent (Poot et al., 2000), we tested for an interaction between histones and FLAG-tagged POLE4, which is present in Polε, but not in CHRAC. As shown in Figure 5B, FLAG-POLE4 strongly IPed endogenous POLE3 from both soluble and chromatin fractions, whereas an interaction between POLE4 and H3-H4 was only observed from DNaseI-digested chromatin, similar to that observed with endogenous POLE3. Importantly, FLAG-tagged POLE3 F44D and POLE4 F74D, which cannot assemble into the Polε complex (as shown in Figure 1), failed to interact with H3 in parallel experiments (Figures S6A and S6B). An interaction between POLE3-POLE4 and H3-H4, but not H2A, was also observed from benzonase-treated chromatin, which excludes indirect interaction via DNA bridging (Figures S6C–S6E). We could also detect interaction with the POLE1 catalytic subunit of Polε, which suggests that interaction with H3-H4 involves the complete Polε complex (Figures S6C–S6E).

To exclude the possibility that the interaction we observed between Polε complex components and histone H3-H4 is indirect via the MCM2 component of the CMG, we IPed POLE3 from the chromatin fraction of cell lines stably expressing MCM2 WT or MCM2 Y81A-Y90A, a mutant that has been shown to lack interaction with histones both *in vitro* and *in vivo* (Huang et al., 2015). To this aim, we expressed exogenous WT and Y81A-Y90A mutant MCM2 while transiently knocking down endogenous MCM2 by siRNA (Figure S6F). In support of a direct POLE3-POLE4 complex interaction with H3-H4, an interaction between endogenous POLE3 and histones H3-H4 was observed in both MCM2 WT- and Y81A-Y90A mutant-expressing cells (Figure S6G). In support of our *in vitro* data, a POLE3ΔC-expressing cell line was unable to bind histones from DNaseI-digested chromatin (Figure 5C). However, POLE3ΔC retained the ability to interact both with POLE4 and POLE1, the catalytic subunit of Polε (Figure S6H).

We also performed HA IPs from HeLa S3 cells stably expressing FLAG-HA-tagged H3.1 and H3.3 (Figure 5D). Endogenous POLE3 and POLE4 IPed with both H3.1 and H3.3, although a stronger interaction was observed with the replicative H3.1 isoform. An interaction with H3.3 raised the possibility that POLE3-POLE4 might be involved in processing of parental histones. To test this hypothesis, we IPed FLAG-tagged POLE4 from DNaseI-digested chromatin and analyzed by western blotting the presence of histone marks specific for newly synthesized or parental histones, including acetylated H4K5 and trimethylated H3K9 and H3K27 (Groth et al., 2007b). An interaction between POLE4 and both parental and newly synthesized histones was detected, suggesting that POLE3-POLE4 might be involved in the processing of both new and parental histones (Figure 5E).

We previously showed that the POLE3-POLE4 complex is able to bind to histone H3-H4 tetramers *in vitro*. To examine this *in vivo*, we followed a sequential IP approach previously reported by Huang et al. to detect endogenous H3 histones in HA IPs from SNAP-HA H3.1-expressing cell lines (Huang et al., 2015; Figure 5F). In accordance with our *in vitro* data, endogenous H3 was observed in sequential IPs from POLE3 FLAG tag-transfected cells but not with FLAG tag controls. These data argue that POLE3 is able to interact with histone tetramers both *in vitro* and *in vivo* (Figure 5F).

### Depletion of the POLE3-POLE4 Complex Affects Chromatin RPA Accumulation and PCNA Unloading

**Figure 6:**
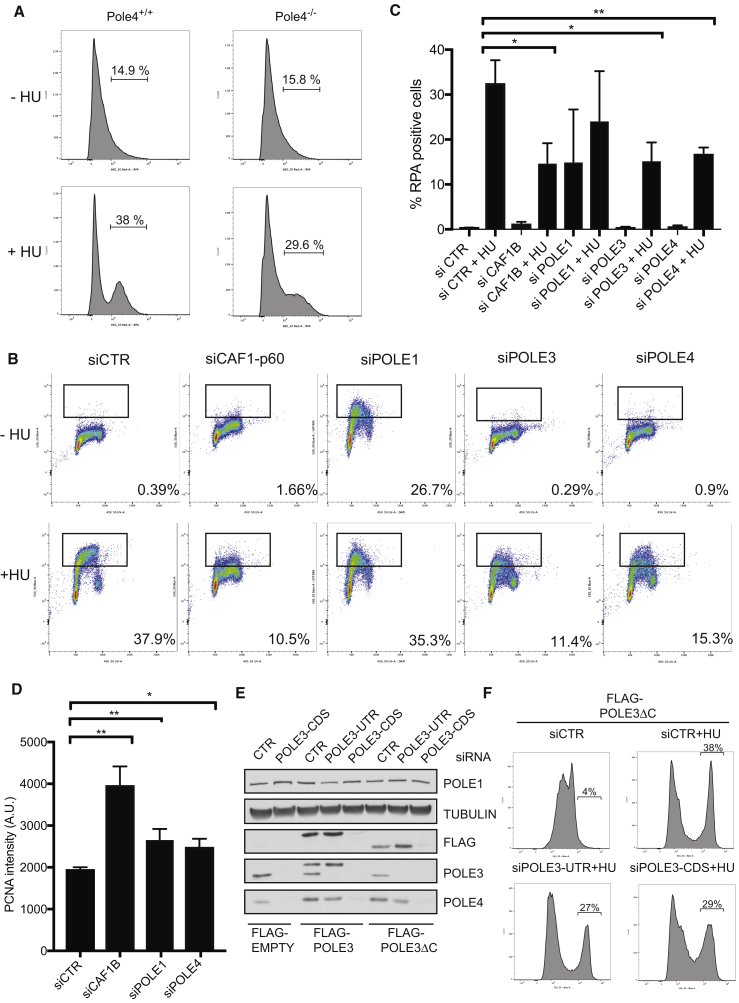
Constitutive or Transient Depletion of the POLE3-POLE4 Complex Affects Nucleosome Disruption/Maturation at the Replication Fork (A) FACS analysis of endogenous chromatin-bound RPA from *POLE4*^+/+^ and *POLE4*^−/−^ MEFs treated or not with 2 mM hydroxyurea for 2 hr. (B) FACS analysis of chromatin from U2OS cells stably expressing GFP-RPA, subjected or not to HU treatment as described in (A). Cells were transfected with siRNAs against the indicated genes, and chromatin purification and FACS analysis were performed 48 hr later. (C) Bar graph showing percentage of chromatin RPA in cells transfected with the indicated siRNA and treated or not with 2 mM HU for 2 hr. Gates for quantification were selected as shown in (B). Biological triplicates (n = 3) are reported with mean, standard deviation, and p value (^∗^p < 0.05, ^∗∗^p < 0.01). (D) Bar graph showing the median of PCNA staining intensity (arbitrary units) from U2OS RFP-PCNA transiently transfected with siRNAs against the indicated genes. Biological triplicates (n = 3) are reported with mean, standard deviation, and p value (^∗^p < 0.05, ^∗∗^p < 0.01). (E) Western blot analysis of cell lysates from HeLa TRex cells expressing empty FLAG, FLAG-POLE3, or FLAG-POLE3ΔC and transfected with the indicated siRNAs in the presence of doxycycline. After SDS-PAGE and nitrocellulose transfer, membranes were incubated with antibodies against the indicated proteins. (F) FACS analysis of endogenous chromatin RPA from HeLa TRex FLAG-POLEΔC cells transfected with the indicated siRNAs and treated or not with 2 mM hydroxyurea for 2 hr.

Defective dismantling of chromatin ahead of the replication fork has been linked to reduced single-strand DNA (ssDNA) and RPA accumulation, likely due to hindrance of the helicase activity of the CMG complex (Groth et al., 2007b, Mejlvang et al., 2014). To examine the possibility that deletion of the POLE3-POLE4 complex might affect single-strand accumulation at the fork, as seen upon Asf1 and CAF-1 knockdown, we analyzed endogenous RPA accumulation, in unchallenged conditions and upon short hydroxyurea (HU) treatment, in mouse embryonic fibroblasts (MEFs) lacking the *POLE4* subunit of the complex (Bellelli et al., 2018). To this aim, we adopted an unbiased fluorescence-activated cell sorting (FACS) approach, which also allowed a parallel analysis of cell cycle parameters (Forment and Jackson, 2015). As shown in Figure 6A, early-passage *POLE4*^−/−^ MEFs showed no significant accumulation of RPA on chromatin in unchallenged conditions. However, when challenged with 2 mM HU for 2 hr, *POLE4*-deficient cells failed to accumulate robust levels of RPA on chromatin when compared to WT MEFs, suggesting the possibility of hindered chromatin disruption ahead of the replication fork.

We recently showed that cells lacking *POLE4* display reduced levels of the Polε complex components POLE1 and POLE2, which we attributed to defective origin activation (Bellelli et al., 2018). Importantly, we also showed that transient deletion of POLE3 and POLE4 in human HeLa cells is not associated with significant reductions in the levels of the other components of Polε complex, while POLE3 and POLE4 are necessary for the stability of one another (Bellelli et al., 2018). To confirm that the effect we observed in *POLE4*^−/−^ MEFs is dependent upon a specific reduction of the *POLE4* subunit, we transiently transfected U2OS cells stably expressing RFP-PCNA and GFP-RPA with siRNAs against POLE1, POLE3, and POLE4 and analyzed chromatin GFP-RPA signal by FACS. As shown in Figures 6B and 6C, depletion of CAF-1B strongly reduced HU-induced RPA accumulation at the replication fork as previously reported (Mejlvang et al., 2014). In untreated conditions, knockdown of POLE1 led to RPA accumulation at the fork, a block in S phase of cell cycle, and reduced BrdU incorporation (Figure S7A). In contrast, transient depletion of POLE3 and POLE4 did not induce significant levels of replication stress nor obvious changes in cell cycle profile or BrdU incorporation (Figure S7A). However, upon HU treatment, cells transiently depleted for POLE3 and POLE4 failed to accumulate robust levels of chromatin RPA when compared to control siRNA-transfected cells. These data suggest that the POLE3-POLE4 complex might affect chromatin disruption/restoration at the replication fork (Figures 6B and 6C).

Groth and collaborators have also reported that reduced chromatin maturation upon CAF-1 knockdown is associated with a corresponding increase in chromatin-bound PCNA due to defective PCNA unloading (Mejlvang et al., 2014). Using a comparable FACS approach, we assessed chromatin levels of exogenous RFP-PCNA and observed a consistent increase in the levels of chromatin-associated PCNA upon transient silencing of POLE1 and POLE4 (Figure 6D). To distinguish between whole chromatin PCNA and PCNA retention specifically at replication forks, we performed iPOND (isolation of proteins on nascent DNA strands) experiments in *POLE4*-deficient cells (Sirbu et al., 2011). As shown in Figure S7B, the levels of PCNA on EdU-labeled DNA were retained upon thymidine release in *POLE4*^−/−^ cells but not in WT cells, consistent with impaired chromatin disruption/maturation at the replication fork.

Finally, we examined the impact of deleting the C-terminal tail of POLE3 (POLE3ΔC) on H3-H4 binding and chromatin dismantling/maturation in cells. To this aim, we knocked down endogenous POLE3 using siRNAs targeting the untranslated region of the POLE3 transcript (siPOLE3 UTR), while expressing FLAG-tagged WT POLE3 or POLE3ΔC mutant at near-physiological levels (Figure 6E). As a control, we used an siRNA targeting the coding sequence of POLE3 (siPOLE3 CDS), which depleted levels of both endogenous and exogenous POLE3 (Figure 6E). In agreement with our previous work, transient knockdown of endogenous POLE3 in FLAG-POLE3-expressing cells did not affect the stability of POLE4, suggesting that FLAG-tagged POLE3 is a functional protein that supports POLE4 stability and full Polε complex formation. Importantly, a similar result was obtained upon endogenous POLE3 knockdown in POLE3ΔC-expressing cells. These data argue that POLE3ΔC is sufficient to maintain POLE4 stability and assembles into the full Polε complex.

To examine the phenotypic consequences of deleting the HBD of POLE3, we performed endogenous chromatin RPA analysis in POLE3ΔC cells upon transient deletion of endogenous and/or exogenous POLE3 WT and mutant proteins. As previously shown, knockdown of endogenous POLE3 reduced RPA accumulation on chromatin upon HU treatment (siPOLE3 CDS). However, expression of POLE3ΔC in cells depleted for endogenous POLE3 (siPOLE3 UTR) failed to rescue RPA accumulation at the fork, suggesting that POLEΔC is defective for Polε-dependent chromatin dismantling/maturation at the replication fork (Figures 6F and S7C). In contrast, POLE3 WT FLAG-tagged protein rescued RPA accumulation at the fork upon HU challenge (Figure S7D). Consistent with compromised nucleosome dynamics at the fork, knockdown of endogenous and exogenous POLE3 in POLE3ΔC cells strongly increased chromatin PCNA levels upon CSK extraction and methanol fixation. However, expression of the exogenous POLE3ΔC protein in the absence of endogenous POLE3 (siPOLE3 UTR) failed to rescue this effect, resulting in higher levels of PCNA, which is again suggestive of defective chromatin maturation (Figure S7E).

Collectively, these data suggest that POLE3ΔC is a separation-of-function mutant that supports POLE3-POLE4 stability and assembly into the Polε complex but is defective for histone H3-H4 interaction and its chromatin disruption/maturation at the fork.

### Transient Knockdown of POLE3-POLE4 Affects Deposition and Recycling of the Replicative H3.1 Histone Variant

**Figure 7:**
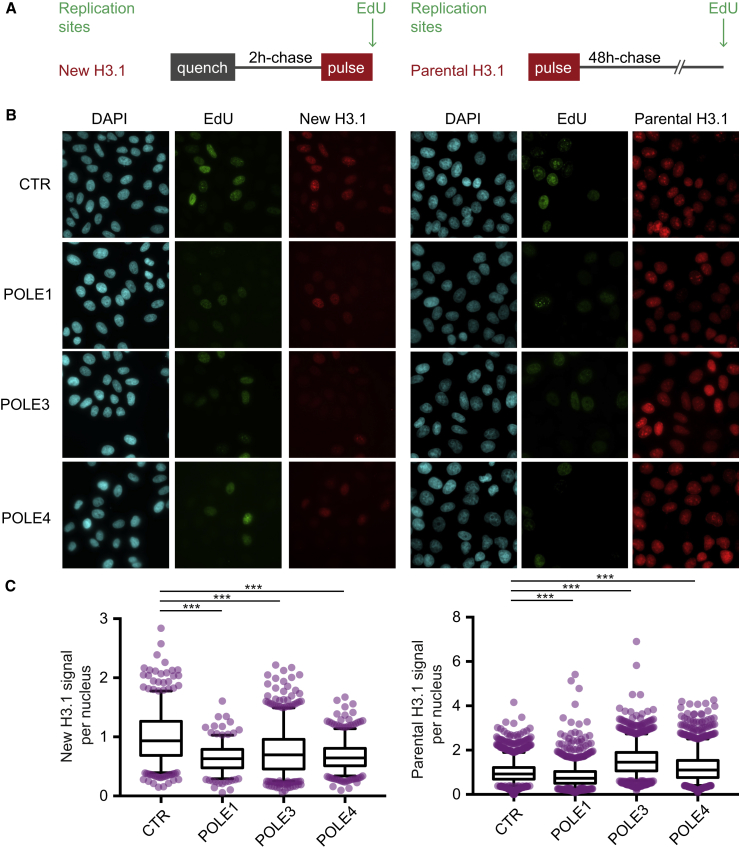
Transient Depletion of the POLE3-POLE4 Complex Affects Histone Deposition in SNAP Tag H3.1-Expressing Cells (A) In HeLa H3.1-SNAP: at left, quench-chase-pulse experiment to follow new H3.1; at right, pulse-chase experiment to follow parental H3.1. A quenching step labels all pre-existing histones with a non-fluorescent dye. A chase step allows synthesis and deposition of new unlabeled H3.1-SNAP. A pulse using the fluorophore TMR (red) labels available H3.1-SNAP. EdU incorporation at the end of the assay allows the detection of replicated DNA (green). A Triton extraction step is performed prior to fixation to eliminate soluble histones and analyze chromatin-bound H3.1. (B) Representative images of new (left) or parental (right) H3.1 (TMR, red) and replication sites (EdU, green) in control, POLE1-depleted, POLE3-depleted, or POLE4-depleted conditions. Scale bars, 10 μm. (C) Quantification of TMR fluorescence signal per nucleus normalized to control mean for new (left) or parental (right) H3.1 in control, POLE1-depleted, POLE3-depleted, or POLE4-depleted conditions (n = 3). For new H3.1 (left), only cells in S phase were quantified.

To examine if POLE3 and POLE4 might affect the main histone deposition pathways in human cells, we exploited the SNAP tag system to specifically monitor deposition of newly synthesized or parental H3.1 histones. The SNAP tag covalently binds to fluorescent or non-fluorescent derivatives of benzylguanine and, when fused to a protein of interest, permits the specific labeling of new or parental subpopulations (Keppler et al., 2003, Jansen et al., 2007). Here, we used a HeLa cell line stably expressing SNAP-HA-tagged H3.1 (Ray-Gallet et al., 2011, Clément et al., 2016), which was subjected to siRNA depletion of POLE1, POLE3, or POLE4 for 48 hr, followed by quench-chase-pulse or pulse-chase experiments to monitor new or parental H3.1, respectively (Figures 7A and 7B). By labeling cells with the BrdU analog EdU, we could distinguish cells in S phase. As expected, we only detected *de novo* deposition of H3.1 in S phase cells (Ray-Gallet et al., 2011). We then quantified the fluorescence signal for new H3.1 and found that it was strongly decreased upon depletion of POLE1, which correlates with the expected reduction in DNA replication upon knockdown of the catalytic subunit of Polε (Figure S6A). Fluorescent signal was also reduced upon knockdown of POLE3 and POLE4, which suggests that transient depletion of the POLE3-POLE4 complex might affect H3.1 deposition at the fork (Figure 7C, left). After knockdown for 48 hr, parental H3.1 was detected in all cells regardless of S phase status. Strikingly, we found that the parental H3.1 signal increased upon POLE3 and POLE4 depletion, compared to the control, while POLE1 depletion led to a slight but significant reduction. These data suggest that POLE3 or POLE4 depletion impacts histone dynamics, possibly due to an imbalance of new versus parental histone deposition on nascent DNA.

## Discussion

A possible role for the leading-strand polymerase Polε in epigenetic inheritance had been proposed based on studies in budding and fission yeast (Iida and Araki, 2004, Tackett et al., 2005, Li et al., 2011, He et al., 2017), but whether this is through a direct role in histone binding and/or an associated chaperone activity was not clear. Here we show that a subcomplex of mammalian Polε, composed of its accessory subunits POLE3 and POLE4, binds directly to histones H3-H4 *in vitro* and *in vivo* and facilitates replication-coupled nucleosome assembly through an intrinsic H3-H4 chaperone activity.

We establish that mammalian POLE3-POLE4 directly binds to H3-H4 in the absence of other replisome components. Our H/D exchange data and confirmatory binding studies argue that the main interaction with H3-H4 involves the C terminus of POLE3 and the histone fold domain of H4 (α2-L2). Indeed, a POLE3ΔC-POLE4 complex lacking the C-terminal α helix of POLE3 (aa 113–140) failed to stably interact with H3-H4 in gel filtration and GST pull-down experiments. Interestingly, H/D exchange experiments, carried out in 300 mM concentrations, also suggested an increased exposure of the central region of H3, which points to a conformational change of histones H3 and H4 upon engagement of H4 by POLE3-POLE4, which is different from that observed with histone chaperones known to bind histone dimers such as NAP1 and DAXX (D’Arcy et al., 2013, DeNizio et al., 2014). Structural studies will be important to define the precise residues in these domains of POLE3-POLE4 and H3-H4 that contribute to the interaction surface and the structural changes associated with co-complex formation.

Importantly, our work establishes that the POLE3-POLE4 complex acts as a bona fide H3-H4 chaperone *in vitro* (De Koning et al., 2007, Hammond et al., 2017). How this activity is coordinated in the context of the full Polε complex and if it cooperates with other chaperones at the fork remains to be determined. MCM2 HBD also stimulates tetrasome formation and supercoiling *in vitro*, in concert with Asf1 (Huang et al., 2015), and is able to bind parental histones *in vivo* (Groth et al., 2007b). Thus, it would be tempting to speculate that MCM2 and POLE3-POLE4 in the context of the full Polε complex might promote histone recycling in a DNA-strand-selective manner, with POLE3-POLE4 chaperone activity directing leading-strand recycling by virtue of its association with the leading-strand polymerase, whereas MCM2 directs recycling on the lagging strand.

Our study also revealed that POLE3-POLE4 binds to histones H3-H4 exclusively in the context of chromatin, which excludes the possibility that they chaperone soluble histones, as has been suggested for MCM2 (Groth et al., 2007b). Interestingly, we observed binding to both histones H3.1 and H3.3, which are deposited in a replication-dependent and -independent manner, respectively (Tagami et al., 2004), although we observe a preference for H3.1 binding *in vivo*. The POLE3-POLE4 complex also binds histones carrying modifications characteristic of both newly synthesized (as marked by H4K5 acetylation) and parental (as marked by H3K9 and H3K27 trimethylation) histones, suggesting that POLE3-POLE4 may handle both parental and newly synthesized histones in the proximity of the leading strand, which would be consistent with its position and function within the replisome.

Constitutive or transient deletion of POLE3-POLE4 in mouse and human cells was found to affect ssDNA formation and nucleosome maturation at the replication fork, a phenotype also seen upon knockdown of several chaperones involved in replication-coupled nucleosome assembly (Groth et al., 2007a, Mejlvang et al., 2014). This effect is uniquely dependent upon histone binding by POLE3-POLE4, since a POLE3ΔC mutant, which supports POLE4 stability and full Polε complex formation, is unable to rescue these phenotypes. We hypothesize that this effect is due to a role for POLE3-POLE4 in handling histones, either new, parental, or both. We tested this hypothesis using SNAP-H3.1-expressing cells and observed a reduction in newly synthesized histone deposition and an increased retention of parental histones. While we cannot exclude an indirect effect on the newly synthesized pathway as observed upon knockdown of the catalytic subunit of the Polε complex, the increased levels of parental histones is specific to POLE3-POLE4 knockdown, which suggests that coordination between the two pathways could be compromised. Thus, we propose that the POLE3-POLE4 complex might act as a coordinator between histone deposition pathways, ensuring a balance between new histone deposition and parental histone recycling. As a leading-strand-specific factor, it might also be required to help deposit histones on the leading strand, as has been recently shown in budding yeast (Yu et al., 2018), or buffer histones at the fork, allowing redistribution to the lagging strand.

We show that POLE3 and POLE4 are able to bind to both H3-H4 dimers and tetramers, despite a likely preference for the tetrameric state. Whether parental H3-H4 tetramers split and/or mix with new H3-H4 during DNA replication has been highly debated. In particular, mass spectrometric studies have shown that parental H3-H4 dimers do not significantly mix with new H3-H4 dimers during DNA replication (Xu et al., 2010). This suggests that parental H3-H4 tetramers could remain stable during their recycling. Conversely, a line of evidence suggests that MCM2 and Asf1 could co-chaperone H3-H4 at the replication fork. In this case, considering that Asf1 handles dimers, parental H3-H4 tetramers might undergo a transient splitting event (Huang et al., 2015, Richet et al., 2015). We envisage that POLE3-POLE4 might directly handle tetramers in an Asf1-independent pathway, or it could function downstream in the MCM2-Asf1 pathway to reassemble H3-H4 tetramers on the leading strand (Petryk et al., 2018, Yu et al., 2018). In future studies, it will be important to understand how these activities are coordinated with Polα, which has been recently shown to function as a chaperone for H2A-H2B (Evrin et al., 2018).

The accurate recycling of parental histones is essential to maintain stemness (Tran et al., 2012), while deposition of newly synthesized histones represents a window of opportunity for cellular commitment and differentiation in multicellular organisms (Nakano et al., 2011). We recently described a complex array of developmental defects, with reduced growth and Seckel-like features, in a mouse model knockout of *POLE4* (Bellelli et al., 2018). Intriguingly, these mice also present with a differentiation defect affecting lymphoid progenitors, leading to T and B cell lymphopenia associated with a relative increase in myeloid precursor-derived blood cells. In addition to this, *Pole4*^−/−^ mice also showed modest anemia, associated with thrombocytosis, which strongly suggests skewed lineage commitment of the hematopoietic stem cell compartment. To our knowledge, this combination of hematological defects is atypical of replication stress mouse models and is rather encountered when epigenetic regulators are inactivated (Rossi et al., 2012, Cedar and Bergman, 2011). Finally, absence of *POLE4* leads to a selective increase in lymphomagenesis (Bellelli et al., 2018). It is therefore more than legitimate to speculate that these phenotypes might be dependent upon an altered epigenetic control of hematopoietic stem cell function, though further work will be needed to molecularly dissect the contribution of chaperone activity of the POLE3-POLE4 complex in this context *in vivo*.

In conclusion, we describe the molecular characterization of the POLE3-POLE4 complex, a previously unappreciated replisome-associated H3-H4 chaperone, which participates in replication coupled-nucleosome assembly. Our findings will prompt future studies to examine the interplay between POLE3-POLE4, MCM2, Polα, and other histone chaperone activities that cooperate to maintain chromatin integrity at the replication fork.

## STAR★Methods

### Key Resources Table

**Table:** 

REAGENT or RESOURCE	SOURCE	IDENTIFIER
**Antibodies**		

Peroxidase-conjugated Goat anti-Mouse IgG (H+L)	Thermo Fisher Scientific	Cat#G-21040; RRID: AB_2536527
Peroxidase-conjugated Goat anti-Rabbit IgG (H+L)	Thermo Fisher Scientific	Cat#G-21234; RRID: AB_2536530
Rabbit Anti-Mouse IgG (H+L) Antibody, Alexa Fluor 546 Conjugated	Thermo Fisher	Cat#A11060; RRID: AB_2534107
Mouse anti-HA	Sigma	Cat#ROAHA Roche; RRID: AB_514505
Rabbit Anti-H4	Millipore	Cat#05-858; RRID: AB_390138
Rabbit Anti-H2A	Millipore	Cat#07-146; RRID: AB_11212920
Rabbit Anti-H4k5ac	Abcam	Cat#Ab51997; RRID: AB_2264109
Rabbit Anti-H3K9me3	Abcam	Cat#Ab8898; RRID: AB_306848
Rabbit Anti-H3K27me3	Abcam	Cat#Ab6002; RRID: AB_305237
Mouse Monoclonal anti-PCNA	Santa Cruz Biotechnology	Cat# sc-56; RRID: AB_628110
Mouse monoclonal anti-Histone H3	Abcam	Cat#ab10799; RRID: AB_470239
Mouse anti-RPA32	Abcam	Cat#ab2175; RRID: AB_302873
Rabbit polyclonal anti-POLE4	Bellelli et al., 2018	N/A
Mouse Monoclonal Anti-POLE2	Abcam	Cat#ab57298; RRID: AB_2166739
Rabbit polyclonal Anti POLE3	Bethyl	Cat#A301-245A; RRID: AB_890598
Rabbit polyclonal Anti POLE1	Genetex	Cat#GTX132100
Monoclonal ANTI-FLAG M2-Peroxidase (HRP) antibody produced in mouse	Sigma	Cat#A8592; RRID: AB_439702
Donkey anti-Mouse IgG (H+L) Highly Cross-Adsorbed Secondary Antibody, Alexa Fluor 488-Conjugated	Thermo Fisher	Cat#A-21202; RRID: AB_141607

**Chemicals, Peptides, and Recombinant Proteins**		

EDTA-free Complete protease inhibitor cocktail	Roche	Cat#COEDTAF-RO
EdU	Thermo Fisher Scientific	Cat#A10044
Biotin-Azide	Thermo Fisher Scientific	Cat#B10184
PhosSTOP phosphatase inhibitor cocktail	Roche	Cat#PHOSS-RO
Streptavidin Sepharose high performance	GE Healthcare	Cat#17-5113-01
CuSO4	Sigma	Cat#PHR1477
Ribonuclease A	Sigma	Cat# R5125
Sodium L-Ascorbate	Sigma	Cat#A7631
Propidium Iodide	Sigma	Cat# P4170
DNase I	New England BioLabs	Cat#M0303S
DAPI	Sigma	Cat#10236276001
Hydroxyurea	Sigma	Cat#H8627
Thymidine	Sigma	Cat#T9250
ANTI-FLAG M2 Affinity Gel	Sigma	Cat#A2220
Anti-HA Agarose	Thermo	Cat#26182
Glutathione-Sepharose 4B	GE Healthcare	Cat# 17-0756-01
Ni Sepharose 6Fast Flow	GE Healthcare	Cat# 17-5318-01
SNAP-Cell TMR-Star	New England Biolabs	Cat#S9106S
SNAP-Cell Block	New England Biolabs	Cat#S9105S
Topoisomerase I	Invitrogen	Cat#38042024
DSS (disuccinimidyl suberate)	Thermo	Cat#21655
Trypsin Gold Mass Spectrometry grade	Promega	Cat#V5280
SYBR Gold Nucleic Acid Gel Stain	Thermo Fisher	Cat#S11494
Prolong Gold antifade reagent with DAPI	Thermo Fisher	Cat#P36935

**Critical Commercial Assays**		

Lipofectamine RNAiMAX	Thermo Fisher	Cat#13778150
Lipofectamine 2000	Thermo Fisher	Cat#11668027
Click-iT EdU Alexa Fluor 488 Flow Cytometry Assay Kit	Thermo Fisher	Cat#C10425

**Experimental Models: Cell Lines**		

Mouse Embryonic Fibroblasts Pole4^−/−^	Bellelli et al., 2018	N/A
HeLa Trex POLE3 WT, POLE3ΔC and F44D mutants	This study	N/A
HeLa Trex POLE4 WT and F74D mutant	This study	N/A
HeLa SNAP-H3.1 and H3.3	Ray-Gallet et al., 2011	N/A
HeLa S3 FLAG-HA-H3.1 and H3.3	Tagami et al., 2004	N/A
U2OS GFP-RPA/RFP-PCNA	Mejlvang et al., 2014	N/A

**Deposited Data**		

Mendeley Dataset	This paper	https://doi.org/10.17632/m5yk53pxt2.2

**Oligonucleotides**		

ON-TARGETplus Non-targeting Control Pool	Dharmacon	Cat#D-001810-10
ON-TARGETplus CAF-1B siRNA	Dharmacon	Cat#L-019937-00-0005
ON-TARGETplus POLE1 siRNA	Dharmacon	Cat#L-020132-00
ON-TARGETplus POLE3 siRNA	Dharmacon	Cat#L-008460-01
ON-TARGETplus POLE4 siRNA	Dharmacon	Cat#L-009850-02
ON-TARGETplus POLE3 siRNA-Individual (siRNA POLE3 UTR)	Dharmacon	Cat#J-008460-10-0005
ON-TARGETplus POLE3 siRNA Individual (siRNA POLE3 CDS)	Dharmacon	Cat#J-008460-11-0005
Custom synthesized siRNA MCM2	Dharmacon	Sense Sequence GGAUGGAGAGGAGCUCAUUUU

**Software and Algorithms**		

Adobe Photoshop CC	Adobe	https://www.adobe.com/es/products/photoshop.html
Adobe Illustrator CC	Adobe	https://www.adobe.com/uk/products/illustrator.html
ImageJ	NIH	https://imagej.nih.gov/ij/
GraphPad Prism 7	GraphPad	https://www.graphpad.com/
FlowJo	TreeStar	https://www.flowjo.com/solutions/flowjo/downloads
Volocity 6.3	PerkinElmer	http://www.perkinelmer.com/lab-products-and-services/cellular-imaging/index.html
CellProfiler	Broad Institute	http://cellprofiler.org/

### Contact For Reagent and Resource Sharing

Further information and requests for reagents should be directed to and will be fulfilled by the Lead Contact, Simon Boulton (simon.boulton@crick.ac.uk).

### Experimental Model and Subject Details

#### Cell lines

Cell lines used in the study are listed in the Key Resources Table. Primary *Pole4*^*+/+*^ and *Pole4*^*−/−*^ MEFs were cultured at 37°C/5% CO_2_/5% O_2_ in Dulbecco’s modified Eagle’s medium (DMEM) (Invitrogen) supplemented with 15% fetal bovine serum (FBS; Sigma) and 1% penicillin-streptomycin (Invitrogen) (Bellelli et al., 2018). Human HeLa TRex cells were cultured in DMEM 10% Tet (Tetracycline)-Free FBS (Clontech) and 1% penicillin-streptomycin while HeLa SNAP-HA-H3.1 and H3.3, HeLa S3 FLAG-HA-H3.1 and H3.3 and U2OS RFP-PCNA/GFP-RPA were cultured in DMEM 10% FBS (Sigma) and 1% penicillin-streptomycin at 37°C/5% CO_2_.

### Method Details

#### Protein expression and purification

The gene encoding for full-length human POLE3 and POLE4 WT and POLE3 and POLE4 fragments were PCR cloned in pGEX 6P-1 (GE Healthcare), containing a GST cleavable tag. POLE3 F44A/D and POLE4 F74A/D mutations were introduced by PCR using Q5 Site-Directed Mutagenesis Kit (NEB), according to manufacturers’ instructions. All proteins used in this study were overexpressed in BL21(DE3) pLysS cell strain (Thermo Fisher Scientific) with exception of untagged human H4 which was expressed in BL21(DE3). Proteins were purified by affinity chromatography, glutathione-Sepharose 4B in a buffer containing 1 M NaCl, 40 mM Tris pH 7.5, 2 mM DTT and 5%–10% glycerol and eluted by inclusion of reduced glutathione in the buffer. GST tag was removed, when specified, with 3C protease before a further size-exclusion chromatography purification step. The genes encoding POLE3 and POLE4 were also PCR cloned via Gateway system (Invitrogen) into pDEST17 for His-tag protein expression and purification via Ni-NTA affinity columns. For generation of the POLE3-4 untagged complex, cells expressing GST-tagged POLE3 and POLE4 were co-lysed. After initial purification on glutathione Sepharose, GST tag was removed by overnight incubation with 3C protease at 7°C. The complex was then captured on heparin column, eluted with a NaCl gradient and polished by size exclusion on HiLoad 16/600 superdex 200 column (GE Healthcare). Histones were expressed and purified according to Luger et al. (Luger et al., 1999).

#### Protein pull-downs

For pull-down of GST-POLE3 and POLE4 WT (as well as GST-POLE3 and POLE4 fragments) with H3-H4 and H2A-H2B complexes, 15 μL of glutathione-Sepharose 4B beads were incubated with GST-tagged proteins for 30 min at 4°C. Histones were there added in a buffer containing 40 mM Tris HCl pH 7.5, 0.25% NP-40 and different NaCl concentrations (as reported in figure legends) for 2 hr at 4°C. For pull-downs of His-tagged POLE3 and POLE4 with histones H3-H4, 15 μL of Ni-NTA beads were resuspended in binding buffer (40 mM Tris pH 7.5, 300 mM NaCl) and incubated with His-tagged proteins for 30 min at 4°C. Histones were then added in the same buffer for 2 hr at 4°C. Streptavidin pull-down of biotinylated H3 and H4 tails (respectively, aa 1-47 and 1-35) incubated with POLE3-POLE4 complex was performed in the same buffer of GST-protein ones. After 3 washes (5-10 min each) in binding buffers pull-downs were resolved by SDS-PAGE and Coomassie staining.

#### Analytical gel filtration of protein complexes

POLE3-POLE4, H3-H4 and H3(EE)-H4, alone or in combination at a final concentration of 100 μM, were analyzed on a Superdex 200 Increase 10/300 column in a buffer containing 20 mM Tris pH 7.5 and 300 mM NaCl. Fractions were collected and analyzed by SDS-PAGE and Coomassie staining.

#### Limited proteolysis

To analyze POLE3-POLE4 complex conformation, limited proteolysis assays were performed by incubating increasing concentrations of Trypsin Gold Mass Spectrometry grade (Promega) (as listed in Figures and Figure legends) in a buffer containing 50 mM Tris-HCl (pH 8) for 10 min at room temperature. Reactions were stopped by addition of 4 x LDS sample buffer (Thermo), resolved by SDS-PAGE and analyzed by Coomassie staining or western blotting against POLE3 and POLE4.

#### Hydrogen/deuterium exchange mass spectrometry (HDX-MS)

Deuterium exchange reactions of POLE3-POLE4, H3-4 and POLE3-POLE4/H3-H4 were initiated by diluting the proteins in D_2_O (99.8% D_2_O ACROS, Sigma, UK) in 20 mM Tris pH 7.5, 300 mM NaCl, 2 mM TCEP to obtain a final D_2_O percentage of ∼91%. For all experiments, deuterium labeling was carried out at 23°C (unless otherwise stated) at five points, 0.3 s (3 s on ice), 3 s, 30 s, 300 s, and 3,000 s in triplicate. The labeling reaction was quenched by the addition of chilled 2.4% v/v formic acid in 2 M guanidinium hydrochloride and immediately frozen in liquid nitrogen. Samples were stored at −80°C prior to analysis. The quenched protein samples were rapidly thawed and subjected to proteolytic cleavage with pepsin followed by reversed phase HPLC separation. Briefly, the protein was passed through an Enzymate BEH immobilized pepsin column, 2.1 × 30 mm, 5 μm (Waters, UK) at 200 μL/min for 2 min, the peptic peptides were trapped and desalted on a 2.1 × 5 mm C18 trap column (Acquity BEH C18 Van-guard pre-column, 1.7 μm, Waters, UK). Trapped peptides were subsequently eluted over 11 min using a 3%–43% gradient of acetonitrile in 0.1% v/v formic acid at 40 μL/min. Peptides were separated on a reverse phase column (Acquity UPLC BEH C18 column 1.7 μm, 100 mm x 1 mm (Waters, UK) and detected on a SYNAPT G2-Si HDMS mass spectrometer (Waters, UK) over a *m/z* of 300 to 2000, with the standard electrospray ionization (ESI) source with lock mass calibration using [Glu1]-fibrino peptide B (50 fmol/μL). The mass spectrometer was operated at a source temperature of 80°C and a spray voltage of 2.6 kV. Spectra were collected in positive ion mode. Peptide identification was performed by MS^e^ (Silva et al., 2005) using an identical gradient of increasing acetonitrile in 0.1% v/v formic acid over 11 min. The resulting MS^e^ data were analyzed using Protein Lynx Global Server software (Waters, UK) with an MS tolerance of 5 ppm. Mass analysis of the peptide centroids was performed using DynamX software (Waters, UK). Only peptides with a score > 6.4 were considered. The first round of analysis and identification was performed automatically by the DynamX software, however, all peptides (deuterated and non-deuterated) were manually verified at every time point for the correct charge state, presence of overlapping peptides, and correct retention time. Deuterium incorporation was not corrected for back-exchange and represents relative, rather than absolute changes in deuterium levels. Changes in H/D amide exchange in any peptide may be due to a single amide or a number of amides within that peptide. The DynamX 3.0 software plots the standard deviation for every peptide. The error band shows the standard deviation of the plotted uptake or difference for each peptide. When there are multiple exposures, as in this experiment, for a given peptide, the maximum standard deviation is plotted for each peptide. A sigma multiplier of 1 is applied to the standard deviation to produce the gray error bar plotted in Figure 3A.

#### DSS cross-linking experiments

10 μM of POLE3-POLE4 and H3-H4 complexes alone or in combination (as explained in figure legends) were incubated in 1 mM DSS (Thermo) for 30 min at 23°C in a buffer containing 20 mM HEPES pH 7.5, 150 mM NaCl (or 300 mM when described), 2 mM DTT. Reactions were then quenched by addition of 50 mM Tris HCl pH 7.5, for 15 min at 23°C. Samples were finally analyzed by SDS-PAGE and Coomassie staining or transferred to nitrocellulose membranes and incubated with antibodies against H3, H4, POLE3, or POLE4.

#### Tetrasome assembly assays

Histone H3–H4 (0.3 μM or increasing concentration from 0.6 up to 2.4 μM) were incubated with equimolar amounts of POLE3-4, POLE3 or POLE4 in reaction buffer containing 10 mM Tris-HCl, pH 7.5, 150 mM NaCl, 0.5 mM EDTA, 10% glycerol and 50 μg/mL BSA at 25°C for 30 min. Afterward, linear dsDNA (Widom 601 sequence, 0.4 μM) was added into each of the chaperone–histone mix and incubated for an additional 60 min at 25°C. Reactions were then resolved using native PAGE (4%–12.0% gradient TBE gel, Invitrogen) followed by SYBR Gold staining (Thermo Fisher).

#### Plasmid supercoiling assay

Histone H3-H4 (1 μM) were incubated with increasing concentrations of POLE3-POLE4 (1-8 μM) in 15 μL reaction buffer containing 10 mM Tris-HCl, pH 7.5, 150 mM NaCl, 0.5 mM EDTA, 10% glycerol and 50 μg/mL BSA at 37°C for 30 min. The circular plasmid, phix174 RF1 DNA was pre-treated with topoisomerase I (Invitrogen, 10 μ/lg DNA) for 30 min and then added (0.4 u μ g in 1 μL) into each of the chaperone-histone mix and incubated for an additional 60 min. Reactions were stopped by adding equal volume of stop buffer (glycerol 25%, 60 mM Tris-HCl, pH 8.0, 30 mM EDTA, 2.0% SDS, 2 μg/μL) followed by further 30 min incubation. Products were resolved by electrophoresis in 1.0% agarose gel followed by SYBR Gold staining.

#### Mouse Embryonic Fibroblasts (MEFs) isolation and culture

*Pole4*^*+/−*^ mice in mixed or C57BL/6 background were mated and pregnant females were subjected to euthanasia under anesthesia at 13.5 days gestation, followed by uterine dissection and individual embryo isolation. Embryo were washed in PBS followed by removal of head (used for embryo genotyping) and internal red organs (heart and liver). The embryo body was minced with sterile razor blades and trypsinized at 37°C for 20 min. Cell suspension was pelleted, resuspended, and plated in 10 cm dishes (now considered passage 0) in DMEM supplemented with 15% FBS (Sigma) and 50 μg/mL penicillin-streptomycin, 2 mM L-glutamine.

#### Generation of HeLa TRex cell lines

HeLa cells expressing FLAG-tag POLE3 and POLE4 WT and mutants were generated using the Flp-In-T-Rex system (Invitrogen) according to manufacturer’s instructions and grown in DMEM supplemented with tetracycline free FBS (Clontech). Cells were transfected using Lipofectamine 2000 (Invitrogen) according to manufacturer’s instructions and selected 48 hr after transfection, in 200 μg/mL Hygromycin (Thermo Fisher Scientific). Protein expression was induced by incubation for 24 hr with doxycycline (final concentration 1 mg/mL).

#### Chromatin isolation and Immunoprecipitation experiments

Chromatin isolation was performed as described in Bellelli et al., 2014. Briefly, cells in mid-exponential phase of growth were washed once in ice-cold 1X phosphate-buffered saline (PBS) and lysed in ice-cold CSK (10 mM PIPES, pH 6.8, 100 mM NaCl, 300 mM sucrose, 1 mM MgCl2, 1 mM EGTA, 1 mM DTT) buffer containing 1 mM ATP and 0.5% Triton X-100 (Pierce Biotechnology) and protease and phosphatates inhibitors (ROCHE) for 10 min on ice. Chromatin-bound and un-bound (soluble fraction) proteins were separated by low speed centrifugation (3,000 rpm, 3 min at 4°C). The pellet (chromatin fraction) was washed in CSK 0.5% Triton, resuspended in CSK 0.1% Triton, 1 mM ATP containing DNase I (1000 U/mL) and incubated for 30 min at 25**°**C. Insoluble undigested chromatin material was removed by high-speed centrifugation. For each fraction, protein amounts deriving from comparable number of cells were used. IP experiments were performed for 2 hr at 4**°**C, using antibodies against endogenous human POLE3 (Bethyl) or FLAG Tag (Sigma) in 0.5% or 0.1% Triton-CSK, for soluble or DNase I digested chromatin respectively.

#### FACS (Fluorescent Acrivated Cell Sorting) analysis

Chromatin FACS analysis of endogenous RPA in *Pole4*^*+/+*^ and *Pole4*^*−/−*^ MEFs (passage 2) and HeLa TRex POLE3 WT or ΔC mutant, as well as exogenous GFP-RPA and RFP-PCNA in U2OS GFP-RPA/RFP-PCNA were performed essentially according to Forment and Jackson, 2015. Briefly, exponentially growing cells (transfected against the listed siRNAs or not) were treated or not with 2 mM hydroxyurea for 2 hr, trypsinized, harvested in media, washed in PBS and permeabilized for 10 min on ice with PBS-Triton 0.2%. After washing in 1% BSA-PBS, cells were fixed with paraformaldehyde 4% at room temperature for 15 min. Chromatin was washed again in 1% BSA-PBS and PBS, stained with DAPI for cell cytometric analysis or further processed for endogenous RPA analysis. In this case, chromatin pellet was blocked in 1% BSA for 30 min and incubated with antibody against RPA (Abcam, see key resource table) for 1 hr at room temperature at 1:200 final concentration. After wash in 1% BSA-PBS and PBS, pellet was incubated with secondary Alexa Fluor-conjugated 488 anti mouse for 30 min at room temperature, washed again in PBS and analyzed using a Flow cytometry analyzer LSRII (Becton Dickinson).

#### Immunofluorescence analysis of chromatin PCNA

For analysis of chromatin PCNA levels, cells were seeded on coverslips and permeabilized for 5 min on ice with CSK-Triton 0.5%, containing protease and phosphatases inhibitors, before being fixed with ice-cold methanol for 20 min at −20**°**C. After PBS washing and permeabilization in PBS-Triton 0.5%, coverslips were blocked in BSA/PBS 1% and incubated with primary anti-PCNA antibody (Santa Cruz Biotechnology, see key resource table) and Alexa Fluor 488 donkey anti-mouse secondary antibody (Invitrogen, see Key Resources Table). Coverslips were washed in PBS and mounted in DAPI containing mounting media (Invitrogen, see key resource table). Images were finally acquired using Zeiss Axio Imager M1 microscope and ORCA-ER camera (Hamamatsu). Image analysis was performed using Volocity 6.3 (Improvision) and CellProfiler softwares.

#### H3.1-SNAP assay *in vivo*

HeLa cells stably expressing H3.1-SNAP-3xHA were previously characterized (Ray-Gallet et al., 2011). Details of SNAP tag assays are described in Clément et al., 2016. For quench-chase-pulse experiments, cells were incubated in complete medium containing 10 μM of SNAP-Cell Block (New England Biolabs) to quench SNAP tag activity, washed twice with PBS, and incubated for 30 min in complete medium to allow SNAP-Cell Block to diffuse out. Cells were then chased for 2 hr in complete medium. Finally, we performed a pulse step by incubating cells for 20 min in complete medium containing 2 μM of SNAP-Cell TMR-Star (New England Biolabs) and 10 μM of EdU (5-ethynyl-20-deoxyuridine). After two washes in PBS cells were incubated again in complete medium for 30 min to allow excess SNAP-Cell TMR-Star to diffuse out. We then performed pre-extraction and fixation. For pulse-chase experiments, cells were incubated in medium containing 2 μM of SNAP-Cell TMR-Star for 20 min, washed twice with PBS, incubated in complete medium for 30 min and washed again twice with PBS. Cells were then incubated in complete medium for a chase time of 48 hr. Finally, cells were washed twice with PBS and reincubated in complete medium containing 10 μM of EdU for 30 min. We then performed pre-extraction and fixation. Pre-extraction and fixation: cells were pre-extracted prior to fixation for 5 min with 0.5% Triton in CSK buffer (10 mM PIPES [pH 7], 100 mM NaCl, 300 mM sucrose, 3 mM MgCl_2_, protease inhibitors), then fixed in 2% paraformaldehyde for 20 min. We performed Click reaction to reveal EdU incorporation (Click-iT EdU Alexa Fluor 488 imaging kit, Invitrogen), stained with DAPI for 5 min and mounted the coverslips in Vectashield. Images were acquired using an AxioImager Zeiss Z1 microscope with a 63x objective.

#### iPOND (isolation of Proteins on Nascent DNA)

iPOND was performed according to standard protocols (Sirbu et al., 2011). *Pole4*^*+/+*^ and *Pole4*^*−/−*^ MEFs were pulse labeled with 10 μM EdU (5-ethynyl-2′-deoxyuridine, Invitrogen) for 10 min. After washing in normal media, cells were released or not in media containing 10 μM thymidine (Sigma) for 5, 10, or 15 min. Cells were then fixed in 1% Formaldehyde (Sigma) in PBS for 20 min at R.T. Crosslinking was subsequently quenched by addition of 0.125 M Glycine for 10 min at R.T. Cells were scraped, pelleted, washed three times in PBS and stored at −80°C. Frozen pellets were resuspended in 0.25% Triton/PBS and incubated at R.T. for 30 min. After washing in ice-cold 0.5% BSA/PBS and PBS, pellets were resuspended in Click reaction cocktail containing 10 μM Biotin-Azide (Invitrogen), 10 mM Na Ascorbate (Sigma) and 2 mM CuSO4 (Sigma) and incubated for 1 hr at R.T. Controls were resuspended in the same buffer containing DMSO instead of Biotin-Azide. After washing in 0.5% ice-cold BSA/PBS and PBS, pellets were then lysed in RIPA buffer (150 mM NaCl, 100 mM Tri pH 7.5, 1% NP-40, 0.1% SDS, 0.5% sodium deoxycolate) containing protease and phosphatase inhibitors (ROCHE) and sonicated using a BIORUPTOR sonicator in 1.5 mL Eppendorf tubes (20-25 cycles at 30 s on, 30 s off setting). Lysates were clarified by high speed centrifugation (13,200 rpm, 15 min at 4°C) and incubated with streptavidin Sepharose beads (GE Healthcare) for 16 hr. After being washed in RIPA and 1M NaCl, beads were resupended in 2X Laemmli buffer, incubated for 25 min at 99°C and loaded on 4%–12% NUPAGE Bis-Tris gels for SDS-PAGE analysis

### Quantification and Statistical Analysis

Statistics, including statistical tests used, number of events quantified, standard deviation standard error of the mean, and statistical significance are reported in figures and figure legends. Statistical analysis has been performed using GraphPad Prism7 software (GraphPad) and statistical significance is determined by the value of p < 0.05.

## Supplemental Information

Document S1. Figures S1–S7

Document S2. Article plus Supplemental Information
