# Supplementary material for: POLE3-POLE4 Is a Histone H3-H4 Chaperone that Maintains Chromatin Integrity during DNA Replication
Source: Mol Cell. 2018 Oct 4;72(1):112–126.e5. doi: 10.1016/j.molcel.2018.08.043 (PMC6179962; doi:10.1016/j.molcel.2018.08.043)
Supplement: Document S1. Figures S1–S7 [file mmc1.pdf]

Molecular Cell, Volume *72*

Supplemental Information

POLE3-POLE4 Is a Histone H3-H4 Chaperone that Maintains Chromatin Integrity during DNA Replication

Roberto Bellelli, Ondrej Belan, Valerie E. Pye, Camille Clement, Sarah L. Maslen, J. Mark Skehel, Peter Cherepanov, Genevieve Almouzni, and Simon J. Boulton

Supplementary Information

POLE3-POLE4 is a histone H3-H4 chaperone that maintains chromatin integrity during DNA replication

Roberto Bellelli[1,], Ondrej Belan[1], Valerie E. Pye[1], Camille Clement[2,3], Sarah L. Maslen[4],J. Mark Skehel[4], Peter Cherepanov[1], Genevieve Almouzni[2,3] & Simon J. Boulton[1]

This file includes Supplementary Figures and legends

Figure S1

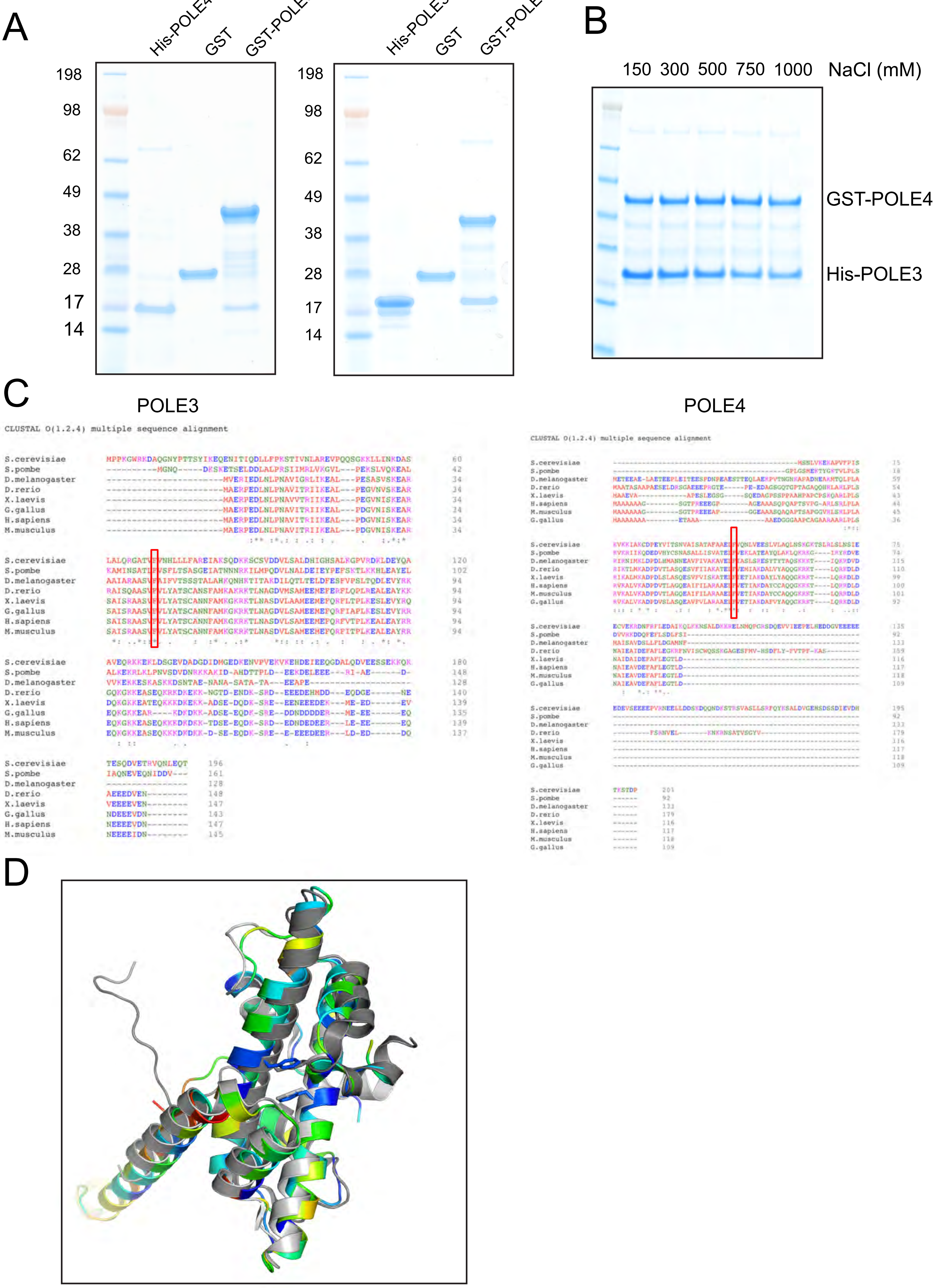
A
His-POLE4 (input)
GST
GST-POLE3
His-POLE3 (input)
GST
GST-POLE4
198
98
62
49
38
28
17
14
B
150 300 500 750 1000 NaCl (mM)
GST-POLE4
His-POLE3
C
POLE3
POLE4
CLUSTAL O(1.2.4) multiple sequence alignment
S.cerevisiae
S.pombe
D.melanogaster
D.rerio
X.laevis
G.gallus
H.sapiens
M.musculus
D

Figure S1: (related to Figure 1)

(A) In vitro GST pull down of the indicated GST-tagged proteins in the presence of His-POLE4 (left) or His-POLE3 (right). (B) Coomassie staining of GST-POLE4/His-POLE3 complex incubated in the described NaCl concentrations. (C) Crustal sequence alignment of POLE3 (left) and POLE4 (right) orthologs in the described species. A red box highlights the highly conserved Phenilalanine residues (F) in position 44 and 74 of POLE3 and POLE4, respectively. (D) POLE3-POLE4 full model, superposed with the Dpb3-Dpb4 S. Pombe structure (pdb 5y26) in grey. Consurf (Landau et al., 2005) was used to colour POLE3-POLE4 residues by conservation from blue (most conserved) to red (least conserved). Yeast POLE4 homologue Dpb3 is reported in dark grey, while yeast POLE3 homologue Dpb4 in light grey. Conserved Phenylalanines are shown in sticks (rmsd 1.5A overall).

Figure S2

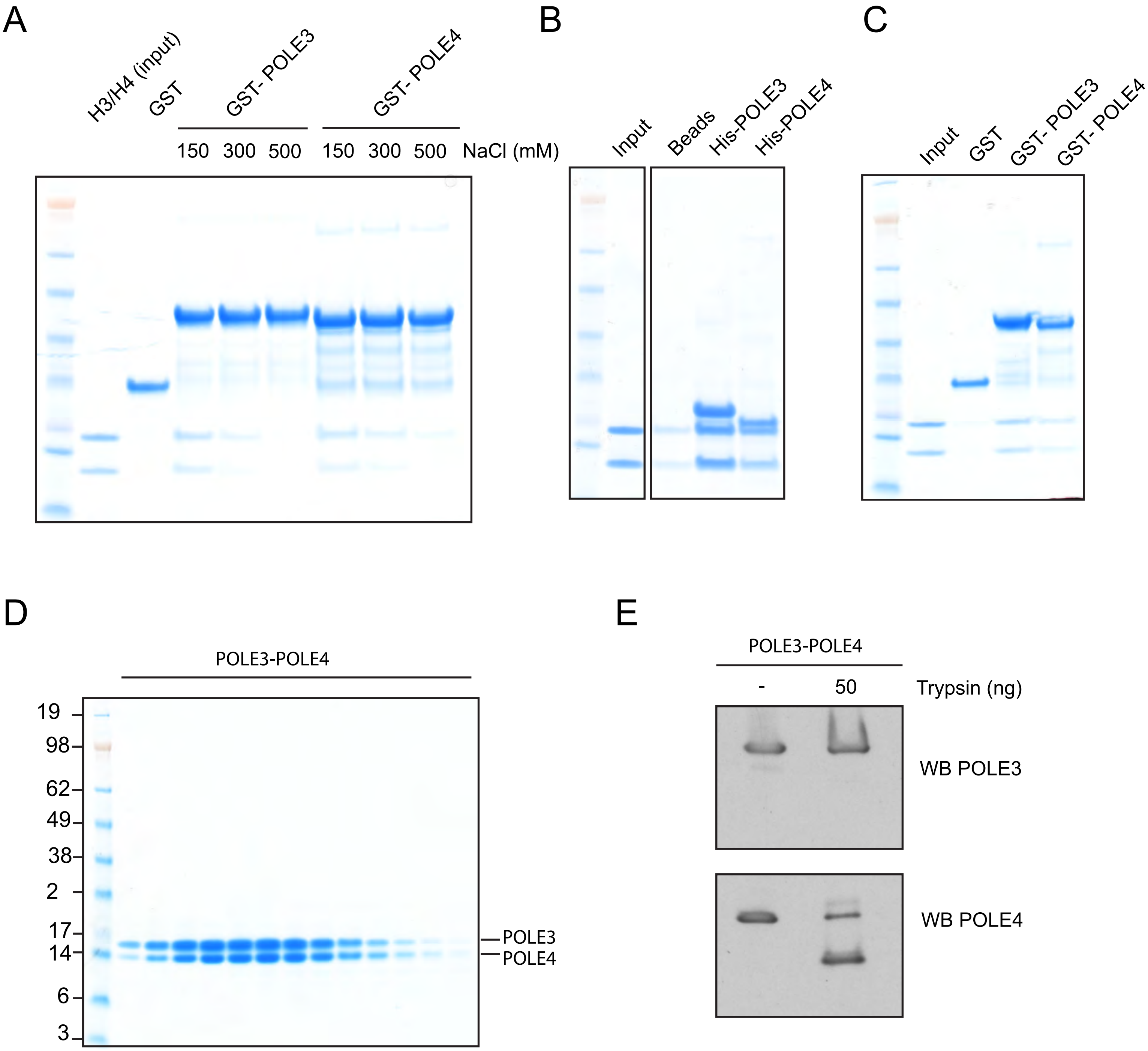
A
H3/H4 (input)
GST
GST- POLE3
GST- POLE4
150 300 500 150 300 500 NaCl (mM)
B
Input
Beads
His-POLE3
His-POLE4
C
Input
GST
GST- POLE3
GST- POLE4
D
POLE3-POLE4
19
98
62
49
38
2
17
14
6
3
POLE3
POLE4
E
POLE3-POLE4
- 50 Trypsin (ng)
WB POLE3
WB POLE4

Figure S2: (related to Figure 2)

(A) *In vitro* GST pull down of the indicated GST-tagged proteins in the presence of H3-H4 and different salt concentrations (150, 300 and 500 mM NaCl) (B) *In vitro* Ni-NTA pull down of the indicated His-tagged proteins in the presence of H3-H4. Ni-NTA beads were used as controls. (C) In vitro GST pull down of the indicated GST-tagged proteins in the presence of the replicative histone variant H3.2-H4 complex. Experiments were performed in 150 mM NaCl. (D) Coomassie staining of gel filtration fractions from three step purification of the untagged POLE3-POLE4 complex used in analytical gel filtrations and H/D exchange experiments. (E) Western blotting analysis of limited trypsin digestion experiments of POLE3-POLE4 complex. After SDS-PAGE, membranes were incubated with antibodies against POLE3 and POLE4.

Figure S3

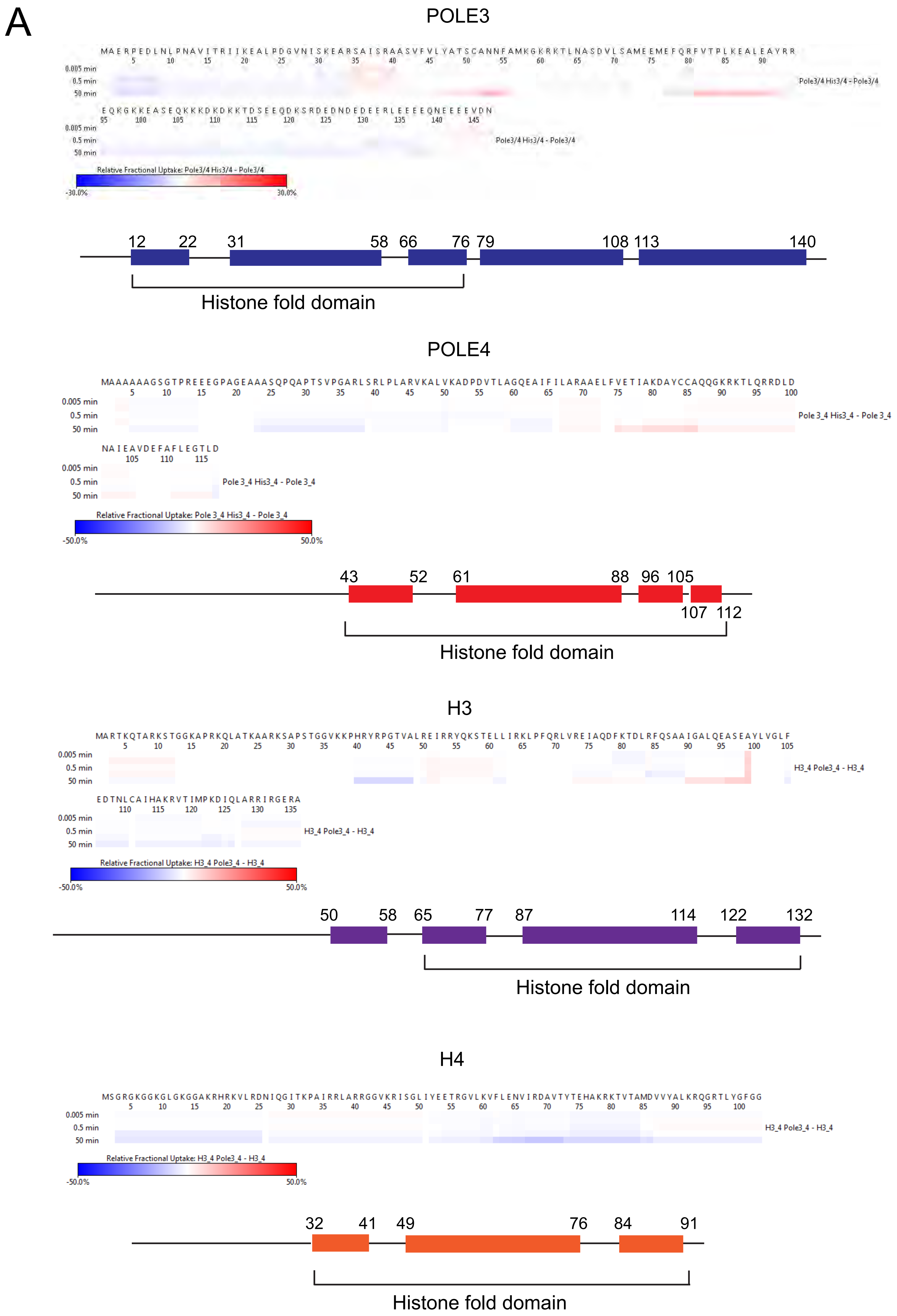
A
POLE3
0.005 min
0.5 min
50 min
Pole3/4 His3/4 - Pole3/4
Relative Fractional Uptake: Pole3/4 His3/4 - Pole3/4
-30.0%
30.0%
12 22 31 58 66 76 79 108 113 140
Histone fold domain
POLE4
Pole 3_4 His3_4 - Pole 3_4
Relative Fractional Uptake: Pole 3_4 His3_4 - Pole 3_4
-50.0%
50.0%
43 52 61 88 96 105
107 112
Histone fold domain
H3
H3_4 Pole3_4 - H3_4
Relative Fractional Uptake: H3_4 Pole3_4 - H3_4
-50.0%
50.0%
50 58 65 77 87 114 122 132
Histone fold domain
H4
H3_4 Pole3_4 - H3_4
Relative Fractional Uptake: H3_4 Pole3_4 - H3_4
-50.0%
50.0%
32 41 49 76 84 91
Histone fold domain

Figure S3: (related to Figure 3)

H/D exchange mass spectrometry heatmaps, indicating differences in deuterium incorporation between POLE3-POLE4 and H3-H4. Regions of increased protection are shown in blue and increased exposure in red. Deuterium labeling was carried out at the indicated three time points in triplicates. Cartoons depicting the respective proteins and domain are indicated at the bottom of the heatmaps.

Figure S4

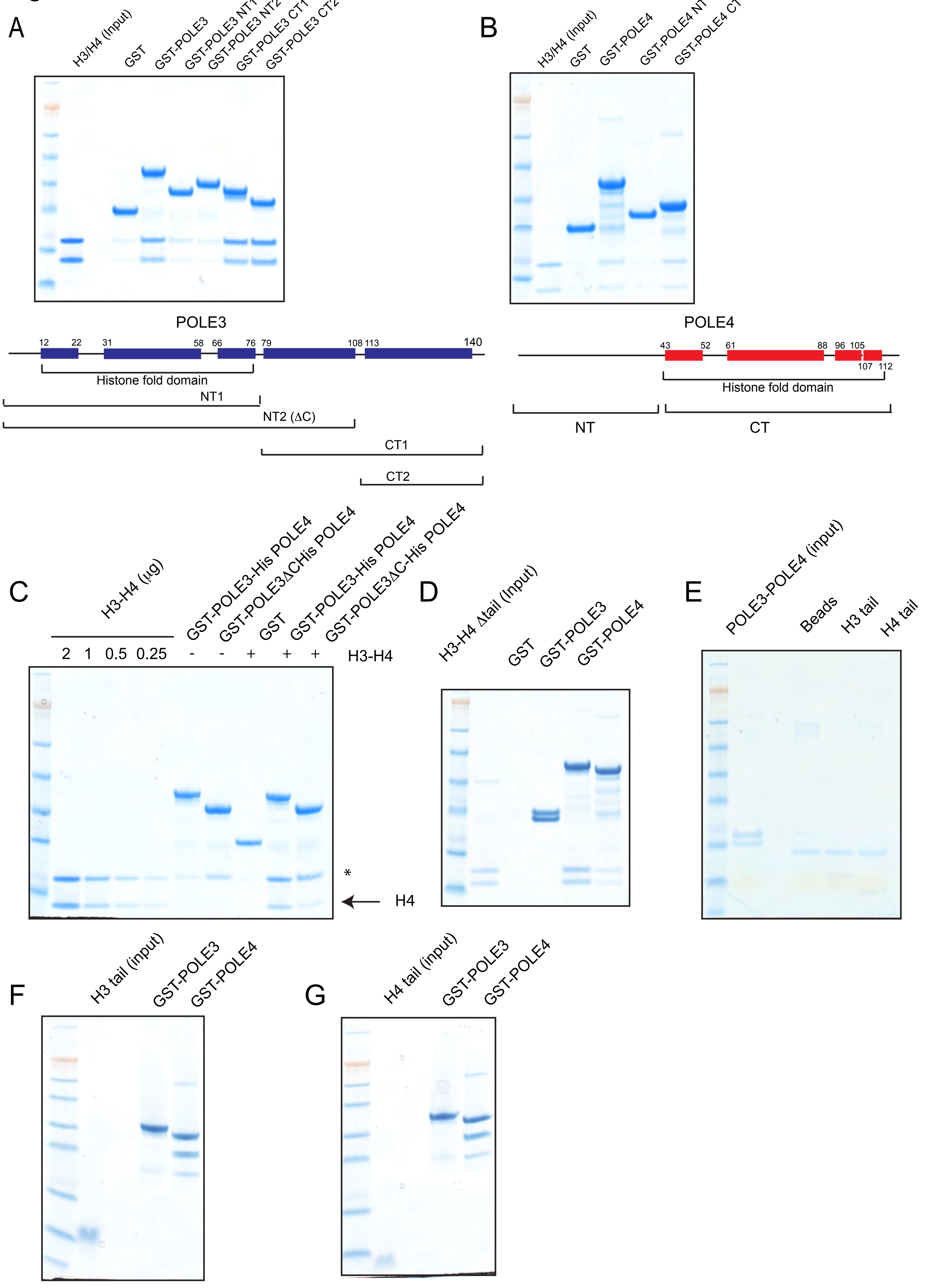

A
H3/H4 (Input)
GST
GST-POLE3
GST-POLE3 NT1
GST-POLE3 NT2 (ΔC)
GST-POLE3 CT1
GST-POLE3 CT2
POLE3
12 22 31 58 66 76 79 108 113 140
Histone fold domain
NT1
NT2 (ΔC)
CT1
CT2
B
H3/H4 (Input)
GST
GST-POLE4
GST-POLE4 NT
GST-POLE4 CT
POLE4
43 52 61 88 96 105 107 112
Histone fold domain
NT
CT
C
H3-H4 (μg)
GST-POLE3-His POLE4
GST-POLE3ΔCHis POLE4
GST
GST-POLE3-His POLE4
GST-POLE3ΔC-His POLE4
2 1 0.5 0.25 - - + + +
H3-H4
*
H4
D
H3-H4 Δtail (Input)
GST
GST-POLE3
GST-POLE4
E
POLE3-POLE4 (input)
Beads
H3 tail
H4 tail
F
H3 tail (input)
GST-POLE3
GST-POLE4
G
H4 tail (input)
GST-POLE3
GST-POLE4

Figure S4: (related to Figure 3)

(A) (B) *In vitro* GST pull down of the indicated GST-tagged proteins in the presence of H3-H4 in 150 mM NaCl concentrations. A Cartoon depicting the fragments of POLE3 and POLE4 used in A and B is also reported in the lower part. (C) GST pull downs of GST-POLE3/His-POLE4 or GST-POLE3ΔC/His POLE4 complexes incubated with H3-H4 in 150 mM NaCl concentrations. Several diluition of H3-H4 (input) are included. Since His-POLE4 and H3 migrate as same molecular weight species, binding in vitro was assessed based on H4 levels in GST-Pull down. For clarity, H4 is indicated by a black arrow while His-Tag POLE4 is indicated by an asterisk (*). (D) In vitro GST pull down of the indicated GST-tagged proteins in the presence of H3-H4Δtail in 150 mM NaCl concentrations. (E) Streptavidin pull down of the indicated biotinylated proteins in the presence of untagged POLE3-4 complex. Beads were used as control. (F) and (G) GST pull down of the indicated GST-tagged proteins in the presence of biotinylated H3 (F) and H4 (G) tails.

Figure S5

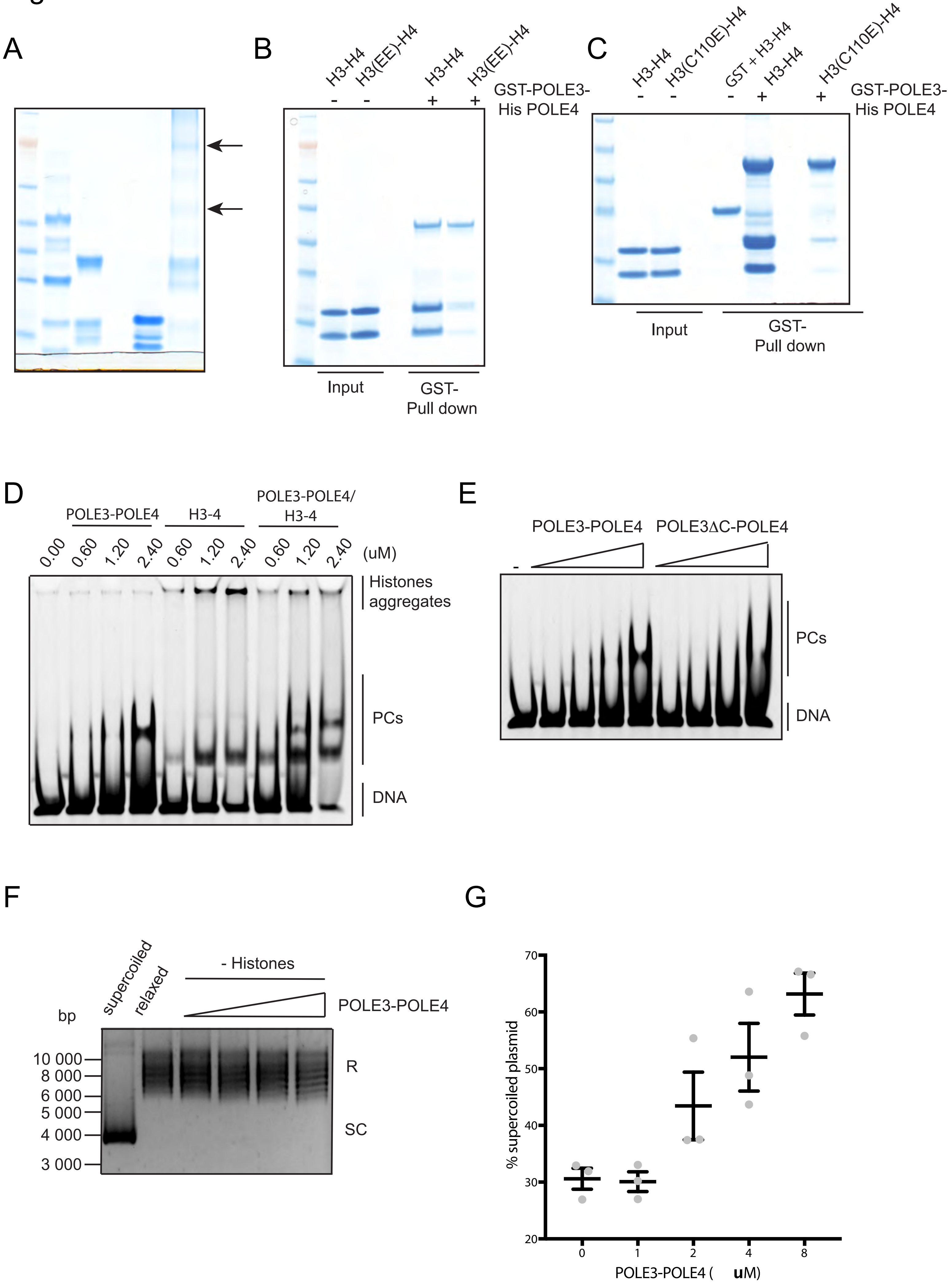
A
B
H3-H4
H3(EE)-H4
H3-H4
H3(EE)-H4
- - + +
GST-POLE3-
His POLE4
Input
GST-
Pull down
C
H3-H4
H3(C110E)-H4
GST + H3-H4
H3-H4
H3(C110E)-H4
- - - + +
GST-POLE3-
His POLE4
Input
GST-
Pull down
D
POLE3-POLE4
H3-4
POLE3-POLE4/
H3-4
0.00
0.60
1.20
2.40
0.60
1.20
2.40
0.60
1.20
2.40
(uM)
Histones
aggregates
PCs
DNA
E
POLE3-POLE4
POLE3ΔC-POLE4
-
PCs
DNA
F
supercoiled
relaxed
- Histones
POLE3-POLE4
bp
10 000
8 000
6 000
5 000
4 000
3 000
R
SC
G
% supercoiled plasmid
70
60
50
40
30
20
0
1
2
4
8
POLE3-POLE4 (uM)

Figure S5: (related to Figure 4)

(A) DSS crosslinking experiments performed in 300mM NaCl. Lanes 4: control without crosslinking; Lanes 1, 2 and 5: samples incubated with 1 mM DSS for 30 min at 23 °C and resolved by SDS PAGE and coomassie staining. Lane 3 was left empty. (B) and (C) GST pull downs of GST-POLE3/His-POLE4 complex incubated with H3-H4 and H3(EE)-H4 (B) or H3(C110E)-H4 (C) mutants in 150 mM NaCl. (D) Tetrasome assembly on linear DNA (Widom 601 sequence) monitored by native PAGE. Lane 1: only DNA; Lanes 2-4: increasing concentrations of POLE3-POLE4 were incubated in the presence of linear DNA; Lanes 5-7: linear DNA incubated with increasing concentrations of H3-H4. Lanes 8-10: linear DNA incubated with increasing concentrations of POLE3-POLE4/H3-H4. (E) Electrophoretic mobility shift assay (EMSA). 0.3 μM Widow sequence dsDNA was incubated with increasing concentrations of either POLE3-POLE4 or POLE3ΔC-POLE4. Protein DNA complexes (PCs) were resolved by native PAGE and stained using SYBR GOLD. (F) Plasmid supercoiling assay resolved by native agarose gel electrophoresis. Lane 1: supercoiled control phix174 RF1 DNA; lane 2: phix174 RF1 DNA relaxed by TOPO I; lane 3-6: phix174 RF1 DNA incubated, in the presence of TOPO I, with increasing concentration of POLE3-POLE4 in the absence of Histones. (G) Quantification of DNA supercoiling assays (n = 3). Mean values of percentage of supercoiled plasmid are reported. Error bars represent standard deviation of the mean.

Figure S6

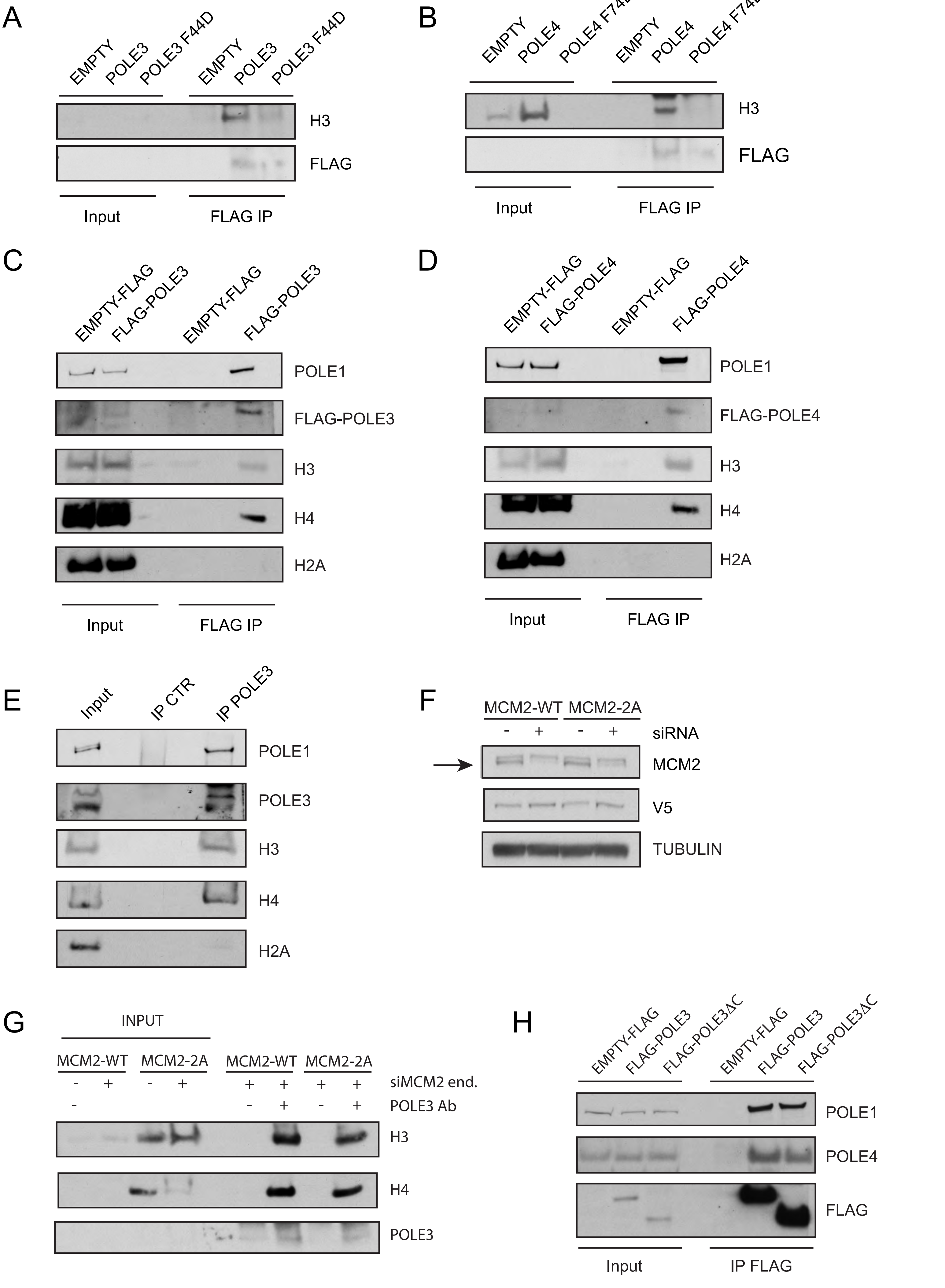
A
EMPTY
POLE3
POLE3 F44D
EMPTY
POLE3
POLE3 F44D
H3
FLAG
Input
FLAG IP
B
EMPTY
POLE4
POLE4 F74D
EMPTY
POLE4
POLE4 F74D
H3
FLAG
Input
FLAG IP
C
EMPTY-FLAG
FLAG-POLE3
EMPTY-FLAG
FLAG-POLE3
POLE1
FLAG-POLE3
H3
H4
H2A
Input
FLAG IP
D
EMPTY-FLAG
FLAG-POLE4
EMPTY-FLAG
FLAG-POLE4
POLE1
FLAG-POLE4
H3
H4
H2A
Input
FLAG IP
E
Input
IP CTR
IP POLE3
POLE1
POLE3
H3
H4
H2A
F
MCM2-WT
MCM2-2A
- + - +
siRNA
MCM2
V5
TUBULIN
G
INPUT
MCM2-WT
MCM2-2A
MCM2-WT
MCM2-2A
- + - + + + + +
siMCM2 end.
- - + - +
POLE3 Ab
H3
H4
POLE3
H
EMPTY-FLAG
FLAG-POLE3
FLAG-POLE3ΔC
EMPTY-FLAG
FLAG-POLE3
FLAG-POLE3ΔC
POLE1
POLE4
FLAG
Input
IP FLAG

Figure S6: (related to Figure 5)

(A) and (B) FLAG immunoprecipitations from HeLa TRex cells expressing the indicated wild type or mutant FLAG-tagged proteins under a tetracycline regulated promoter. Cells were induced with doxycicline for 24 hours and lysed in CSK-Triton 0.5%. FLAG-IPs were performed on DNAseI digested chromatin fractions; after SDS-PAGE and western blotting, membranes were incubated with antibodies against H3 and FLAG. (C) and (D) FLAG immunoprecipitations from HeLa TRex expressing empty-FLAG and FLAG-tagged POLE3 (C) or POLE4 (D) under a tetracycline regulated promoter. Cells induced with doxycicline were lysed in CSK-Triton 0.5%. After chromatin solubilisation with benzonase, FLAG-IPs were performed followed by SDS-PAGE and western blotting with antibodies against the indicated proteins. (E) Immunoprecipitation of endogenous POLE3 from human HeLa cells performed after CSK-Triton 0.5% extraction and benzonase chromatin digestion. After SDS-PAGE and nitrocellulose transfer, membranes were incubated with antibodies against the indicated proteins. (F) Western blot analysis of U2OS cells stably expressing V5-tagged MCM2-WT or 2A mutant (Y81A Y90A) and transfected or not with siRNA against endogenous MCM2. (G) Immunoprecipitation of endogenous POLE3 from U2OS cells stably expressing V5-tagged MCM2-WT or 2A mutant (Y81A Y90A) and transfected or not with siRNA against endogenous MCM2. After SDS-PAGE and nitrocellulose transfer, membranes were incubated with antibodies against the indicated proteins. (H) FLAG immunoprecipitations from HeLa TRex cells expressing Empty-FLAG, FLAG-POLE3 or FLAG-POLE3ΔC under a tetracycline regulated promoter. Cells, induced with doxycicline for 24 hours, were lysed in CSK-Triton 0.5% and FLAG-IPs were performed on soluble fraction; after SDS-PAGE and western blotting, membranes were incubated with antibodies against POLE1, POLE4 and FLAG.

Figure S7

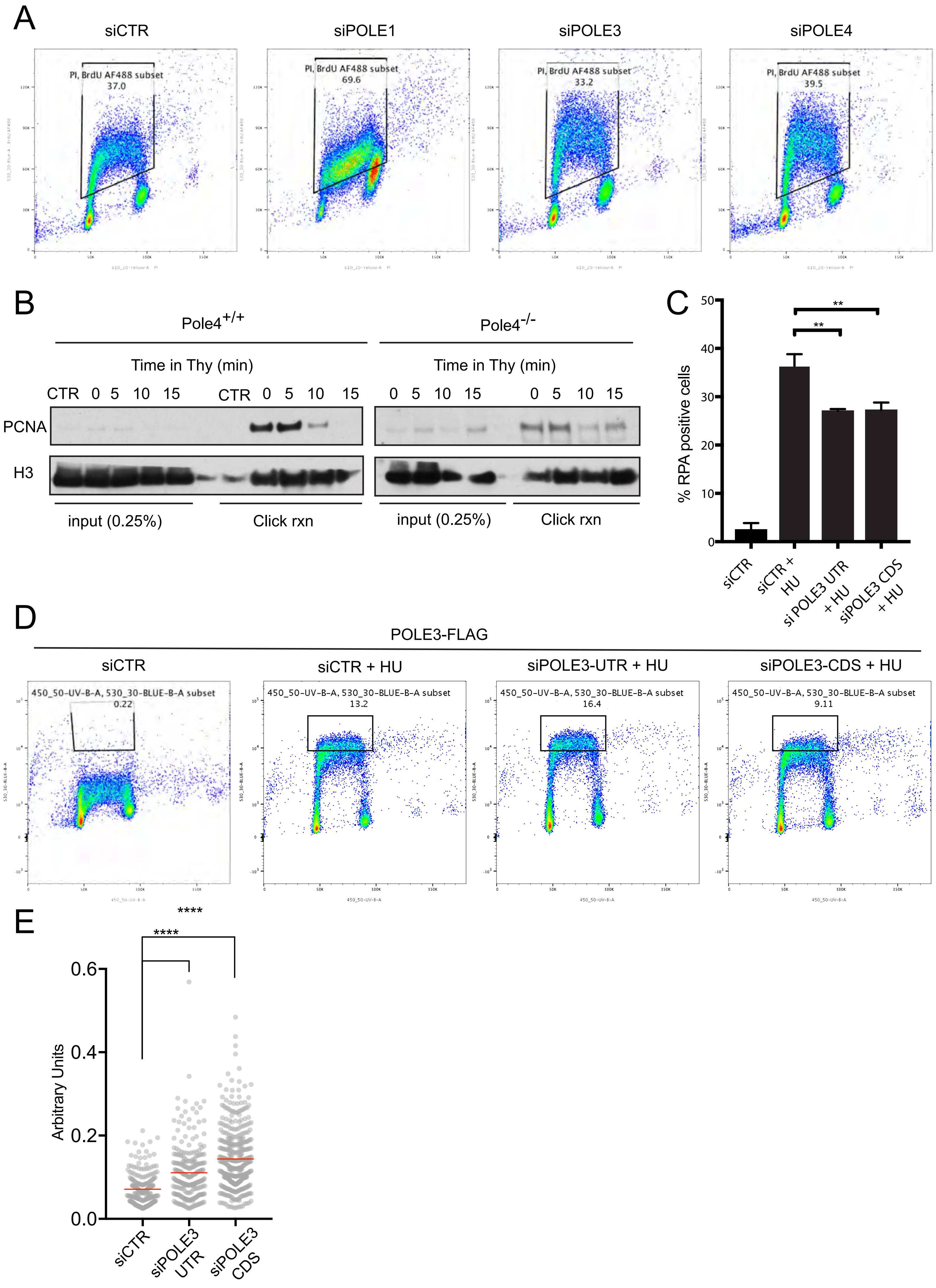
A
siCTR
siPOLE1
siPOLE3
siPOLE4
PI, BrdU AF488 subset
37.0
69.6
33.2
39.5
B
Pole4+/+
Pole4-/-
Time in Thy (min)
CTR 0 5 10 15 CTR 0 5 10 15
0 5 10 15 0 5 10 15
PCNA
H3
input (0.25%)
Click rxn
C
% RPA positive cells
**
siCTR
siCTR + HU
si POLE3 UTR + HU
siPOLE3 CDS + HU
D
POLE3-FLAG
siCTR
siCTR + HU
siPOLE3-UTR + HU
siPOLE3-CDS + HU
450_50-UV-B-A, 530_30-BLUE-B-A subset
0.22
13.2
16.4
9.11
E
****
Arbitrary Units
0.6
0.4
0.2
0.0
siCTR
siPOLE3 UTR
siPOLE3 CDS

Figure S7: (related to Figure 6)

(A) BrdU and Propidium Iodide FACS (Fluorescence Activated Cell Sorting) analysis of HeLa cells transfected for 48 hours with siRNA against the indicated proteins. (B) iPOND (isolation of Protein on Nascent DNA) experiments performed on *POLE4*$^{+/+}$ and $^{-/-}$ MEFs. Cells were pulse labelled with EdU for 10 min and released in Thymidine for the indicated times before being processed for iPOND. (C) Bar-graph showing percentage of high level chromatin RPA in FLAG-POLE3ΔC cells transfected with the indicated siRNA and treated or not with 2mM HU for 2 hours. Biological triplicates (n = 3) are reported with mean, standard deviation and p-value (**p<0.01). (D) FACS analysis of endogenous chromatin RPA from HeLa TRex cells expressing FLAG-POLE3 under a tetracycline regulated promoter, trasfected with the indicated siRNAs and subjected or not to HU treatment. (E) Bar-graphs showing mean and standard deviation of PCNA staining intensity (arbitrary units) from HeLa TRex cells expressing FLAG-POLE3ΔC under a tetracycline regulated promoter and transfected with the indicated siRNAs (***p<0.001; n = 3).
